# The deSUMOylase SPF2 and the cohesin regulators SGO2 and CTF18 suppress crossovers near centromeres

**DOI:** 10.1038/s41477-026-02329-1

**Published:** 2026-07-24

**Authors:** Rigel Salinas Gamboa, Joiselle Blanche Fernandes, Qichao Lian, Dipesh Kumar Singh, Stephanie Durand, Divyanshu Sahu, Laia Capilla-Perez, Hernan Lopez, Alexandra Kalde, Raphael Mercier

**Affiliations:** 1 Department of Chromosome Biology, Max Planck Institute for Plant Breeding Research, Cologne, Germany; 2 Guangdong Basic Research Center of Excellence for Precise Breeding of Future Crops, Guangdong Laboratory for Lingnan Modern Agriculture, Guangdong Provincial Key Laboratory for the Development Biology and Environmental Adaptation of Agricultural Organisms, South China Institute for Soybean Innovation Research, College of Life Sciences, South China Agricultural University, Guangzhou, China; 3 Present Address: Laboratory of Plant Reproduction, Center for DNA Fingerprinting and Diagnostics (CDFD), Hyderabad, India

**Keywords:** Plant genetics, Genetics, Plant reproduction

## Abstract

Crossovers (COs) ensure proper chromosome segregation during meiosis and generate genetic diversity. COs are non-uniformly distributed along chromosomes and almost universally suppressed in centromere-proximal regions, notably creating an important bottleneck for plant breeding. The mechanism of this CO suppression is still not fully understood, but the chromatin state is a contributing factor. Here we identify three factors that actively limit proximal CO in *Arabidopsis thaliana*: the cohesion establishment factor CTF18, the centromeric cohesin protector SGO2 and the deSUMOylase SPF2. The mutation of these factors allows both the formation of COs in the centromere-proximal region where they were completely absent in the wild type and the enhancement of their frequency where they were rare. COs can be further increased by combining these mutations together or with mutation in the DNA methylase *CMT3*, suggesting that multiple mechanisms prevent proximal COs in parallel. The identification of the very conserved CTF18, SGO2 and SPF2 as suppressors of centromere-proximal COs highlights the importance of cohesin turnover in this process and opens up new possibilities for plant breeding.

## Abstract (web summary)

Gamboa et al. identified three proteins that restrict meiotic crossovers near centromeres. This finding suggests that cohesion regulation plays a role in this suppression and creates opportunities to unlock recombination for plant breeding

## Main

Centromeres are specific regions of chromosomes where the kinetochore complex attaches, enabling microtubule binding and chromosome segregation during mitosis and meiosis^1^. In many eukaryotes, centromeres contain the histone variant CENH3/CENPA and can be small as a single nucleosome as in budding yeast, a few kilobases (kb) long as in fission yeast or constituted of several megabases (Mb) of satellite arrays like in humans (171 bp alpha-satellite) and *Arabidopsis thaliana* (CEN178 satellite)^2,3^. In many species, centromeres are surrounded by regions with low gene density and high transposon density, rich in DNA methylation and heterochromatin marks, and with suppressed meiotic recombination^3–5^. These centromere-proximal regions are commonly referred to as pericentromeres. However, there is no consensus in the literature on defining the boundaries of pericentromeres, either considering gene density, DNA/histone methylation levels or reduced recombination rates^3–8^.

Meiotic crossovers (COs) enhance genetic diversity and are also essential for accurate chromosome segregation at meiosis^9–12^. By creating exchanges between homologous chromosomes, COs physically link them and promote their balanced segregation. Meiotic COs result from the repair of programmed DNA double-strand breaks (DSBs)^9–12^. Only a small subset of DSBs is repaired as homologous COs; the rest is repaired using either the sister chromatid as a template or the homologous chromatid but resulting in non-CO events^9–12^. The distribution of COs along chromosomes is non-uniform, with regions prone to CO and other regions where they are rare or absent, most notably in the pericentromeric regions^13–17^. CO suppression at the centromere-proximal region, the so-called ‘centromere effect’, was initially described in *Drosophila*^18^ and is conserved across species, from unicellular to multicellular eukaryotes, including yeast, plants and animals^19,20^. CO suppression applies to the centromere itself (for example, a satellite array) and extends to the flanking pericentromeric DNA at varying distances between species. In budding yeast with a point centromere, CO suppression ranges in different chromosomes from ~1 to 18 kb (ref. ^21^). In rice, *A. thaliana*, *Drosophila* and humans, the region lacking COs extends from a few 100 kb to a few megabases beyond the centromere^16,22–26^. In many crop species, the pericentromeric CO suppression extends to more than half of the chromosomes, representing tens of megabases of DNA in tomato or maize, and 100 Mb in wheat^4,27–30^.

This centromere-proximal CO suppression is thought to be beneficial for fertility, as COs near centromeres have been associated with an increased risk of chromosome mis-segregation in humans, *Drosophila* and yeast^2,31–34^. It is suggested that CO and thus chiasmata formation closer to the centromere would locally weaken sister chromatid cohesion, which is essential for balanced sister chromatid distribution at second meiotic division. Alternatively, proximal COs could generate an entanglement preventing chromosome separation at anaphase I. However, these hypotheses cannot easily explain the extension of CO suppression to large proportions of the chromosomes.

Increasing centromere-proximal COs could positively impact plant breeding. As described above, in many crops, large regions spanning megabases of DNA near centromeres are devoid of COs^4,35–38^. Even if gene density tends to be reduced, the centromere-proximal region contains genes that are thus inaccessible to breeders. For example, 18% of the barley genes are located in the low-recombining centromere-proximal region (20-fold lower than the genome average)^5,39^. The wheat pericentromeric region contains crucial defence response genes^4,40^. The tomato mosaic virus resistance gene (*Tm-2*^2^) was introgressed from a wild species into commercial varieties, together with a 64 Mb non-recombining pericentromeric chromosomal segment that alters multiple tomato traits^41–44^. Low COs in centromere-proximal regions thus challenge breeding by limiting the combination of favourable alleles and counterselection of harmful ones.

The centromere effect was first described nearly a century ago^18^, yet the mechanisms underlying CO suppression are still not fully understood and are being explored in various organisms. In *A. thaliana*, pericentromeres are enriched in heterochromatin marks including DNA methylation, lysine 9 of histone H3 methylation (H3K9me2) and histone variant H2A.W which participate in CO suppression^15,45,46^. Similarly, mutations in the H3K9 methyltransferase genes in *Drosophila* (*Su(var)3-9*) and fission yeast (*lr4*) increase centromere-proximal DSBs and COs^47,48^. In *A. thaliana*, disruption of the H3K9 methyltransferase KYP/SUVH4 SUVH5 SUVH6, or the non-CG DNA methyltransferase CMT3, or the histone variant H2A.W also leads to an increase in pericentromeric COs^15,45–48^. However, the absence of the CG methylation factor MET1 in *A. thaliana* has the opposite effect, a decrease in centromere-proximal COs^15,49^. This contrasting effect may be a result of the differences in the heterochromatin mark H3K9me2, which is reduced in *cmt3* mutants but remains intact in *met1* mutants^50,51^. Besides chromatin factors, kinetochores and cohesins have been implicated in CO suppression in yeast. In budding yeast, disruption of subunits of the inner kinetochore complex Ctf19/CCAN increases both COs and non-crossovers in pericentromeres^52^. Ctf19 plays a dual role in suppressing centromere-proximal COs by limiting DSB formation and by recruiting and enriching the Rec8–cohesin complex, which in turn prevents the repair of DSBs as COs^52–54^. In fission yeast, DSB formation depends on the Rec8–Rec11 cohesin complex, which tends to be replaced by the Rec8–Psc3 complex in the pericentromeric region, preventing DSBs and thus CO formation^2,55^. This enrichment of Rec8–Psc3 in the centromere-proximal region is dependent on the H3K9me-binding protein Swi6 (ref. ^2^). Finally, the transverse element of the synaptonemal complex (which zips the homologues together during meiotic prophase), Zip1, was shown to contribute to pericentromeric CO suppression in budding yeast^52^.

In this Article, we conducted a genetic screening in *A. thaliana* and identified three factors that contribute to the suppression of centromere-proximal COs: the cohesion establishment factor CTF18, the centromeric cohesion protection factor Shugoshin 2 (SGO2) and the deSUMOylase SPF2. In each corresponding mutant, we observed an increase in pericentromeric COs, including regions where COs are absent in the wild type (the non-recombining zone (NRZ)). This establishes the role of cohesin regulators in preventing COs near centromeres in plants. We also show that proximal COs can be further increased by combinations of these mutations and disruption of *CMT3*, suggesting that multiple mechanisms contribute to CO suppression in parallel. Unlocking centromere-proximal COs does not significantly affect fertility, suggesting a promising strategy for accessing genes near centromeres in plants.

## Results

### Identification of mutants with increased centromere-proximal COs

**Fig. 1:**
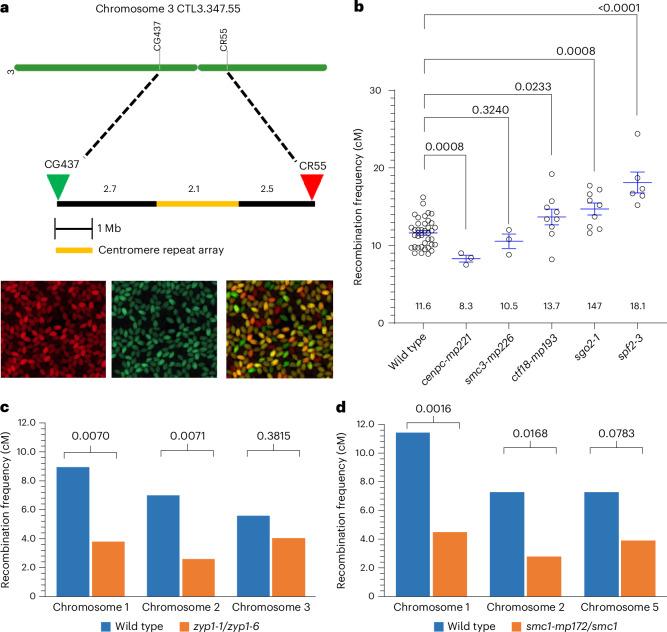
Testing candidate mutations for elevated COs in pericentromeres. **a**, The physical interval on chromosome 3 defined by the transgene conferring green (CG437) and red (CR55) fluorescence to seeds in the line CTL.3.437.55. Physical sizes are indicated in Mb. Seeds show segregation of the GFP and dsRED fluorescence. **b**, Measure of recombination frequency in centimorgans in the CTL.3.437.55 interval. Each dot represents a measurement on ~500 seeds. Blue lines indicate the mean of the recombination frequency ± standard error of the mean (s.e.m.). The mean is also indicated along the *x* axis for each genotype. Wild-type samples from different populations (that is, sister plants of different mutants) are pooled together. Measures for individual populations are provided in Extended Data Fig. 1. *P* values are from two-sided Mann–Whitney tests (*n* = 38, 3, 3, 9, 9 and 6, respectively). **c**,**d**, Recombination frequencies (cM) measured in 3 centromeric intervals in *zyp1* (Col/L*er*) (**c**) and *smc1* (Col/Ws) (**d**) hybrids. Schematic representation of centromeric intervals is provided in Extended Data Fig. 1. *P* values are Fisher’s exact tests. Source data

**Extended Data Fig. 1:**
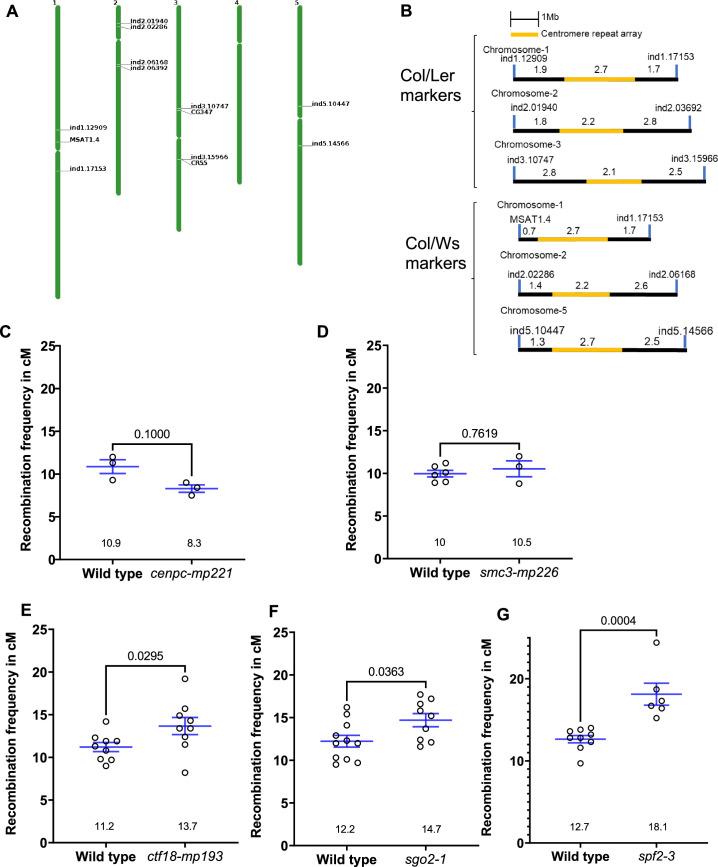
Measurement of recombination in individual populations. (**A**) Graphical representation of the genomic position of the markers used to measure recombination in CTL.3.437.55, Col/L*er* and Col/Ws. (**B**) Schematic representation of the physical intervals defined by the markers used to measure recombination in Col/L*er* and Col/Ws hybrids. Physical sizes are indicated in Mb. (**C-G**) Related to Fig. 1b. Measure of recombination frequency in centimorgans (cM) in the CTL.3.437.55 interval. Each dot represents a measurement on ~500 seeds. Blue lines indicate the Mean of the recombination frequency +/- standard Error (SEM) and the value (cM) is given for each genotype. Each mutant is compared to wild type from the same segregating population (sister plants). Statistical tests are two-sided Mann-Whitney tests. Source data is provided. Source Data

Aiming to identify factors that limit COs in the centromere-proximal regions, we selected candidate mutants (see below) and measured recombination rates in intervals flanking centromeres using two methods. In the first method, we used fluorescent tagged lines (FTLs), also known as traffic lines, containing two genetically linked transgenes that express fluorescent proteins in seeds (green fluorescent protein (GFP) and dsRed). Scoring seed fluorescence allows measuring recombination in the interval defined by the position of the two transgenes^56^ (Fig. 1a). We used one well-characterized FTL line, CTL3.437.55, which contains the transgenes CG437 (Col_CEN Chr3: 10,942 kb) and CR55 (Chr3: 18,255 kb), defining an interval of 7.3 Mb spanning the centromere of chromosome 3, including a 2.1 Mb *CEN178* satellite array^8^ (Fig. 1a). We crossed candidate mutants with CTL3.437.55 and selected plants from the F_2_ populations that were heterozygous for the fluorescent transgenes and either wild type or homozygous for the tested mutation. Recombination was measured based on the segregation of fluorescence in seeds from the self-fertilization of mutant and wild-type sister plants. In the second method, when candidate mutations were available in two different strains, we measured recombination in hybrids (Col/L*er* or Col/Ws) by PCR-genotyping genetic polymorphisms flanking centromeres (Extended Data Fig. 1a,b) in F_2_ progeny of F_1_ hybrids, either wild type or homozygous for the tested mutation.

One landmark of meiosis is the orientation of sister kinetochores toward the same pole at meiosis I, which allows the distribution of homologous chromosomes rather than sister chromatids^57^. Our group identified factors that promote the monopolar orientation of kinetochores at meiosis in *A. thaliana* through a forward genetic screening^58^. This screen uncovered mutant alleles of genes encoding cohesin subunits (SMC1, SCC3, SMC3, REC8), cohesion establishment factors (CTF18, DCC1), protectors of centromeric cohesion (SGO2), an inner kinetochore protein (CENP-C) and a deSUMOylase (SPF2)^58^ (Supplementary Fig. 1). While monopolar orientation and/or cohesion are weakened in these mutants, correct chromosome segregation is maintained. We reasoned that the changes in the organization of the centromere-proximal region that affect kinetochore orientation in the identified mutants may also affect CO formation near centromeres. We selected at least one mutant for each of the functional categories described above (presumably null mutant of *CTF18*, *SGO2* and *SPF2*, hypomorphic mutants of the essential genes *CENPC*, *SMC1* and *SMC3*) and tested them for modified recombination in one to three intervals spanning a centromere (Fig. 1). In addition, we tested a mutation (*zyp1*) that affects the synaptonemal complex, as this structure was shown to contribute to the suppression of centromere-proximal COs in other species^7,52,59^. The results for all the tested mutants are shown in Fig. 1.

**Extended Data Fig. 2:**
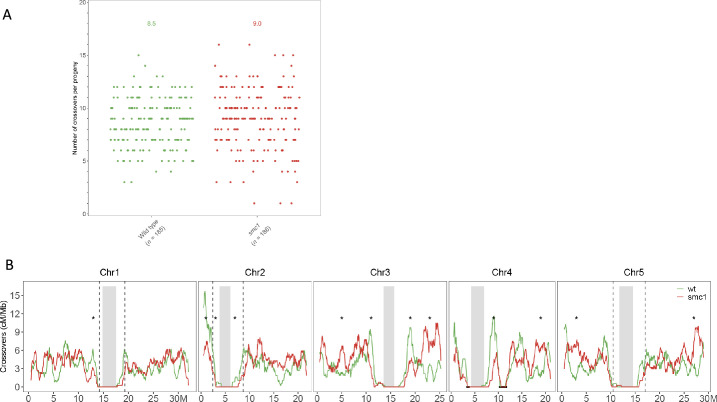
Crossover are redistributed in *smc1-mp172/smc1-1*. (**A**) Total CO number per F2 plant. Each dot represents the CO number in an F2 plant. The average COs per F2 is indicated for each genotype. Both wild type and mutant are Col/Ws-4 Hybrids. (**B**) CO distribution on all 5 chromosomes in wild type (green) and *smc1-mp172/smc1* (red). The y-axis corresponds to the CO frequency (cM/Mb) using a 1 Mb window size and 50 kb step size. The x-axis corresponds to physical distance in Mb in the Col-CEN v1.2 assembly. The grey shading indicates the centromeric *CEN178* repeats array. Asterisks indicate 2 Mb non-overlapping intervals with a significantly different recombination rate than wild type (corrected two-sided Mann-Whitney tests, * p < 0.05). The exact p values are provided in the source data. The vertical dotted lines in black indicate the borders of the intervals used in Fig. 1d. The CO numbers in the three defined intervals are as follows (*n* in wild type/*n* in *smc1/P* value Fisher’s exact test): Chr1 9/2/0.036; Chr2: 27/7/0.003; Chr3 20/7/0.0095. Source Data

Among the mutants affected in monopolar orientation were *cenpc-mp221*, a point mutation in CENP-C, a core kinetochore subunit^58,60,61^. We presume that the *cenpc-mp221* mutation only slightly affects the CENP-C function, as growth and development appear unaffected, while CENP-C is likely essential in *A. thaliana*, as in other eukaryotes^58,62^. In *cenpc-mp221*, we observed reduced recombination in the tested interval on chromosome 3 when compared to the wild type (Fig. 1b and Extended Data Fig. 1c), contrasting with previous observations in *Saccharomyces* *cerevisiae* kinetochore mutants^52^. Next, we tested two alleles of cohesin subunits, *smc1-mp172* and *smc3-mp226* (ref. ^58^). SMC1 and SMC3 are essential subunits of the cohesin complex, which, together with SCC1/REC8 and SCC3, form a ring-shaped structure that holds together sister chromatids at mitosis and meiosis and organizes chromatin folding^63–65^. These alleles are also hypomorphic as SMC1 and SMC3 in *A. thaliana* are essential for plant development^66,67^. Recombination was not significantly modified in *smc3-mp226* in the CTL3.437.55 interval (Fig. 1b and Extended Data Fig. 1d). In *smc1-mp172/smc1*, two of the three tested intervals showed significantly reduced recombination, while the third interval appeared reduced but not significantly (Fig. 1d). We thus extended the study of *SMC1* by performing whole-genome sequencing of *smc1* F_2_ progeny and the respective wild-type control (*n* = 185 wild-type Col/Ws-4 and *n* = 186 *smc1-mp172/smc1*) (Extended Data Fig. 2). The total CO number was slightly increased in the mutant (9 versus 8.5, Mann–Whitney test *P* = 0.02), and COs were shifted from proximal towards more distal regions, in accordance with the interval analysis (Fig. 1d and Extended Data Fig. 2). The CO peaks observed on both sides of all pericentromeres in the wild type were reduced in *smc1*. This shows that SMC1 contributes to the shaping of the CO landscape by promoting CO in proximal regions. Thus, the *cenpc*, *smc1* and *smc3* alleles tested here do not support a role for the kinetochore or the cohesin subunits in limiting pericentromeric COs.

In yeast and drosophila, the transverse/central element of the synaptonemal complex contributes to the suppression of centromere-proximal COs^7,52,59^. However, mutation of *ZYP1*—the transverse element in *A. thaliana*—leads to a significantly lower CO frequency in two of the three centromeric intervals that we tested (Fig. 1c). This is consistent with genome-wide data showing distal redistribution of COs in *zyp1* mutants^68^ and suggests that ZYP1 does not contribute to suppressing pericentromeric COs.

**Fig. 2:**
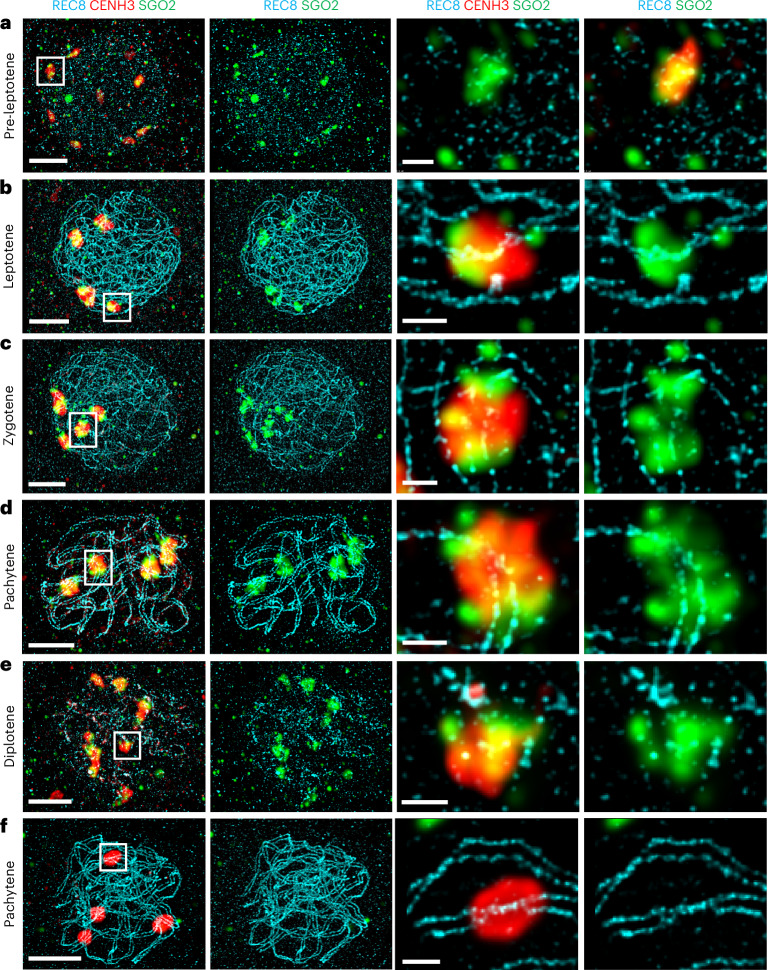
SGO2 localizes at pericentromeres throughout the meiotic prophase. Immunostaining of the chromatin axis protein REC8 (blue, all columns), the centromeric cohesin protector SGO2 (green, all columns) and the centromeric histone variant CENH3 (red, columns 1 and 3) in male meiotic cells at prophase I. **a**–**e**, Wild type: pre-leptotene (**a**), leptotene (**b**), zygotene (**c**), pachytene (**d**) and diplotene (**e**). **f**, *sgo2-1* pachytene. For each panel, the two first images show the entire nucleus (scale bars, 3 µm), while the two last images display magnified views of a selected region indicated by a box in the first image (scale bars, 0.5 µm). Throughout prophase, SGO2 protein is detected in the pericentromeric regions adjacent to the centromeric CENH3. The signal is not detected in the *sgo2-1* mutant, showing the specificity of the antibody. Each experiment was performed at least three times.

Next, we investigated loss-of-function mutants of CTF18 and SGO2 that also support monopolar orientation of sister kinetochores at meiosis I^58^. CTF18 is a conserved replication factor that favours the establishment of sister chromatid cohesion^69–71^. Its absence at replication may affect subsequent events, including recombination. Shugoshins (SGOs) are conserved protectors of centromeric cohesins^72^. *A. thaliana* SGO1 and SGO2 are partially redundant for this function, protecting centromeric cohesion at anaphase I^73,74^. Neither SGO2 nor CTF18 is necessary for normal plant growth and development (Supplementary Fig. 2)^71,72^. We raised an antibody against SGO2, revealing a pericentric signal at meiosis, which is absent in the *sgo2* mutant (Fig. 2). SGO2 predominantly localizes at pericentromeres, flanking the CENH3 protein that decorates the centromere. SGO2 is present in prophase, which is compatible with a potential role in regulating CO formation at pericentromeres. In both *ctf18* and *sgo2*, we observed a ~20% significant increase in recombination in the CTL3.437.55 interval compared to the wild type (Fig. 1b, Extended Data Fig. 1e,f and Source Data). This suggests that the cohesin regulators CTF18 and SGO2 contribute to the suppression of pericentromeric COs.

Finally, we tested another mutant affecting monopolar orientation, a loss-of-function allele of SPF2 (ref. ^58^). SPF2 is one of the two *A. thaliana* homologues of the yeast deSUMOylase Ulp2, which notably regulates cohesin turnover. SPF2 is not required for plant development (Supplementary Fig. 2)^75,76^. In *spf2*, recombination was increased by ~50% compared to wild-type controls in the CTL3.437.55 interval (18.1 cM (centimorgans) versus 11.6 cM) (Fig. 1b and Extended Data Fig. 1g). This suggests a central role for the deSUMOylase SPF2 in the restriction of centromere-proximal COs.

In brief, we identified three genes: *CTF18*, *SGO2* and *SPF2*, whose loss-of-function mutations led to an increase in CO frequency in the pericentromeric CTL3.437.55 interval, which is particularly marked in *spf2*. This suggests that CTF18, SGO2 and SPF2 negatively regulate CO formation in the pericentromeres and motivated a comprehensive study of the distribution of CO in their absence.

### Genome-wide analysis of COs in *ctf18, sgo2* and *spf2*

**Extended Data Fig. 3:**
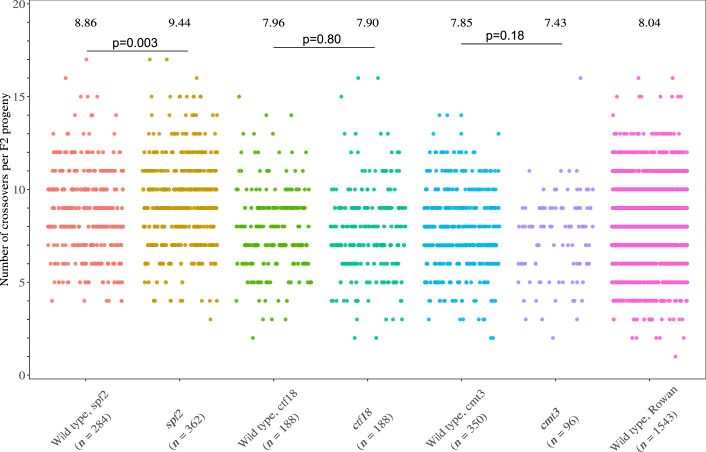
Total CO number per F2 in wild type and mutants. Related to Fig. 3. Total CO number per F2 in wild type and mutants. Each dot represents the CO number in an F2 plant and the average COs per F2 is indicated for each genotype. Mutants *spf2* and *ctf18* are compared to wild type from the same population (sister plants). The *cmt3* and corresponding wildtype data are from Underwood et al.^15^. The large wildtype population is from Rowan et al.^77^. P values are two-sided Mann-Whitney tests. Source data is provided. Source Data

To assess the impact of *ctf18*, *sgo2* and *spf2* on COs across the genome, we generated novel loss-of-function mutant alleles in a second strain (L*er*) using CRISPR-Cas9 (clustered regularly interspaced short palindromic repeats and CRISPR-associated protein 9) (Supplementary Fig. 1). We crossed Col and L*er* heterozygous mutants to create *ctf18*−/−, *spf2*−/− and *sgo2*−/− Col/L*er* F_1_ hybrids, which were reproduced by self-fertilization. Wild-type +/+ plants were selected in the same F_1_ populations and also reproduced by selfing. The resulting F_2_ plants were sequenced by Illumina to detect COs using Col/L*er* polymorphisms. To gain power, we combined our 472 wild-type F_2_ plants with datasets from previous studies using the same Col/L*er* hybrid, making a total of 2,365 F_2_ plants (Extended Data Fig. 3)^15,77^. In addition, previous studies showed that a mutation in *CMT3* or *H2A.W6/7* increases centromere-proximal COs^15,45^. We reanalysed the available *cmt3*, *h2a.w.6*, *h2a.w.7* and h*2a.w.6 h2a.w.7* raw sequence data, which are also Col/L*er* F_2_ samples, with the same parameters and reference genome^15,45^. Finally, we included data from *recq4a recq4b*, in which CO are increased ~4-fold genome-wide, making RECQ4 the strongest generic anti-CO factor described to date^78–80^.

**Fig. 3:**
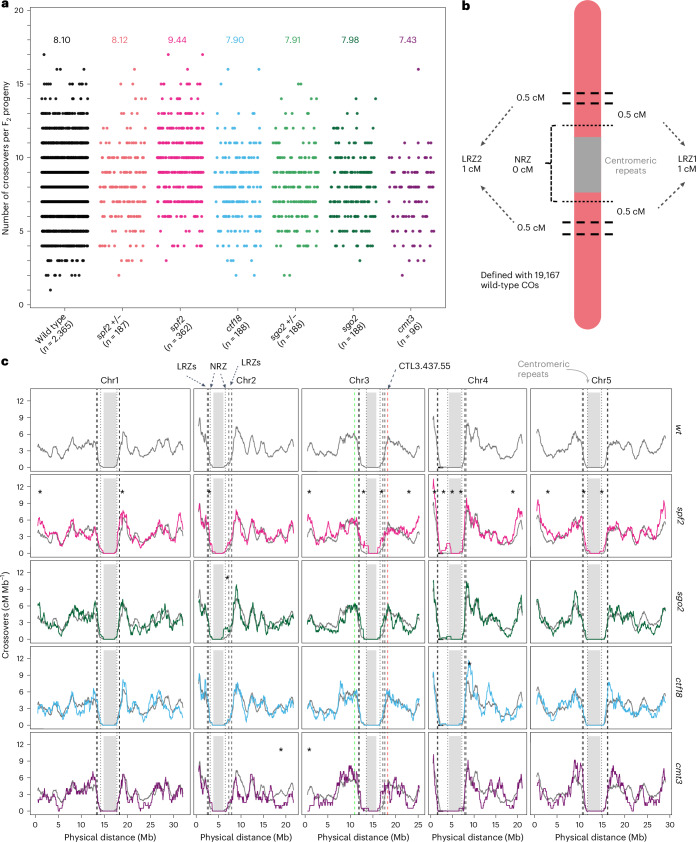
Distribution of COs along chromosomes at the megabase scale. **a**, Total CO number per F_2_ plant. Each dot represents the CO number in an F_2_ plant. The average CO per F_2_ is indicated for each genotype. **b**, Schematic representation of NRZ, LRZ1 and LRZ2 on the physical chromosome. The centromeric repeat array is indicated in grey. **c**, CO distribution on all 5 chromosomes in wild type (grey), *spf2* (pink), *sgo2* (green), *ctf18* (blue) and *cmt3* (purple). The *y* axis corresponds to the CO frequency (cM Mb^−1^) using a 1 Mb window size and 50 kb step size. The *x* axis corresponds to physical distance in Mb in the Col-CEN v1.2 assembly. The grey shading indicates the centromeric *CEN178* repeats array, and the vertical dotted lines indicate the borders of the NRZ, LRZ1, LRZ2 (black) and fluorescent markers (green and red). Asterisks indicate 2 Mb non-overlapping intervals with a significantly different recombination rate than wild type (corrected two-sided Mann–Whitney tests, *P < 0.05). The exact *P* values are provided in the source data. Source data

**Fig. 4:**
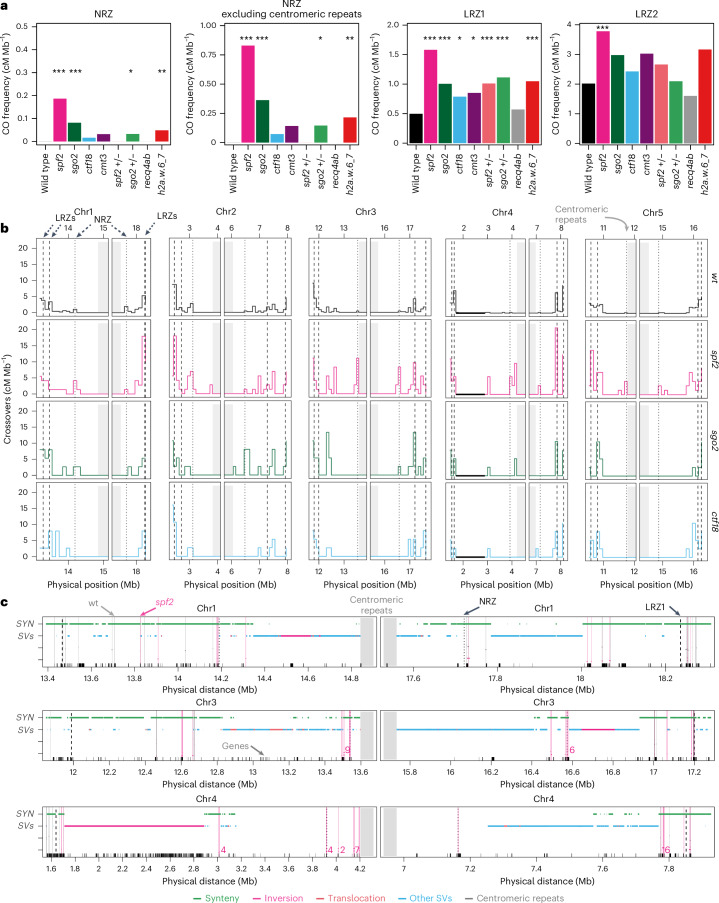
CO frequencies are increased in NRZ and LRZ in *spf2*, *sgo2*, *ctf18* and *cmt3*. **a**, CO frequency (cM Mb^−1^) in the NRZ, in the NRZ excluding the CEN178 repeat arrays, in the LRZ1 and in the LRZ2. The *y* axis shows the CO frequency (cM Mb^−1^), merging the five chromosomes. Stars correspond to two-sided Fisher exact tests with FDR correction (*P < 0.05, **P < 0.01, ***P < 10^−3^). The exact *P* values are provided in the source data. Data on individual chromosomes are shown in Extended Data Fig. 5. **b**, CO distribution in the centromeric region (NRZ and LRZs). CO frequencies are measured in non-overlapping 100 kb windows in wild type (grey), *spf2* (pink), *sgo2* (green) and *ctf18* (blue). The *y* axis represents CO frequencies (cM Mb^−1^), and the *x* axis shows physical position (Mb). The grey shading represents the centromeric repeat array and the vertical dotted lines the NRZ, LRZ1 and LRZ2 borders. The black horizontal line on chromosome 4 indicates the well-described inversion between Col and L*er* genomes. **c**, High-resolution distribution of COs in NRZ and LRZ1 in wild type and *spf2* on chromosomes 1, 3 and 4. Other chromosomes and genotypes are shown in Supplementary Figs. 4 and 5. The *x* axis corresponds to physical distance in Mb. Note that the repeat array (grey shading) is truncated. Black ticks on the *x* axis represent genes. Vertical lines indicate the position of COs detected in wild type (grey) and *spf2* (pink). When multiple COs occurred at overlapping positions, the counts are indicated. Horizontal bars indicate Col/L*er* syntenic regions (green), inversions (pink), translocations (red) and other structural variants (blue). Source data

First, we defined the regions where COs are suppressed in the wild type (Fig. 3b). The NRZ was previously defined as a contiguous region flanking the centromere where no COs are observed in the wild type^8^. As we included more samples than in the previous study, we slightly redefined the NRZ borders (Table 1) (19,167 COs in 2,365 F_2_, equivalent of 4,730 gametes). This NRZ encompasses the centromeric repeat arrays, which are completely devoid of COs (0/4,730 gametes). Note that even if the arrays of centromeric repeats do not contain usable markers, a CO in these regions would be detected because of the genotype switch of the flanking markers (for example, Col/Col on one side and Col/L*er* on the other side). None of the 19,164 COs identified in 4,730 gametes were mapped to the centromeric repeat regions, suggesting that COs are extremely rare or absent in the *CEN178* repeat arrays that represent a total of 12.6 Mb of DNA, 9.6% of the genome. The NRZ extends beyond the centromere repeats for all five chromosomes, adding 3.7 Mb of CO-free DNA. The NRZ represents a total of 16.3 Mb or 12.3% of the genome (Figs. 3 and 4 and Table 1). Flanking the NRZ, we designated two low-recombining zones (LRZs), LRZ1 and LRZ2, being a physical non-overlapping interval, each corresponding to a genetic size of 1 cM in wild type (0.5 cM on each side of the centromere) (Fig. 3b). From centromere outwards, LRZ1 immediately flanks the NRZ, and LRZ2 flanks LRZ1. Altogether, the LRZ1 regions of the five chromosomes span 9.8 Mb (and, by definition, 5 cM), with a CO rate of 0.5 cM Mb^−1^, an ~8-fold reduction compared to the rest of the genome (non-NRZ/LRZ). LRZ2 regions span 2.41 Mb with a CO rate of 2 cM Mb^−1^, an ~2-fold reduction compared to the rest of the genome, and might be considered at the edge of the centromere effect (Table 1 and Fig. 4a).

**Table 1:** Properties of the NRZ and the LRZs

	Border position (Mb)								Size (Mb)						CO rate (cM Mb^−1^)				
	Centromere repeats, left	Centromere repeats, right	NRZ, left	NRZ, right	LRZ1, left	LRZ1, right	LRZ2, left	LRZ2, right	Chromosome	Centromere repeats	NRZ	LRZ1	LRZ2	Whole genome		NRZ1	LRZ1	LRZ2	Non-NRZ/LRZ
Chr1	14.84	17.56	14.19	17.72	13.47	18.24	13.29	18.26	32.5	2.72	3.53	1.24	0.20	3.04		0.00	0.81	5.02	3.52
Chr2	3.72	5.95	3.11	6.47	2.71	7.27	2.45	7.92	22.2	2.22	3.36	1.20	0.90	3.25		0.00	0.83	1.11	4.19
Chr3	13.60	15.73	13.54	16.57	11.99	17.20	11.81	17.63	25.7	2.14	3.03	2.18	0.61	2.93		0.00	0.46	1.63	3.69
Chr4	4.20	6.98	3.92	7.16	1.64	7.85	1.54	8.12	21.6	2.77	3.24	2.97	0.37	3.07		0.00	0.34	2.70	4.29
Chr5	11.78	14.55	11.75	14.87	10.81	16.13	10.60	16.24	29.5	2.77	3.11	2.20	0.32	3.13		0.00	0.45	3.10	3.79
All									131.6	12.62	16.28	9.79	2.41	3.08		0.00	0.51	2.07	3.83

**Extended Data Fig. 4:**
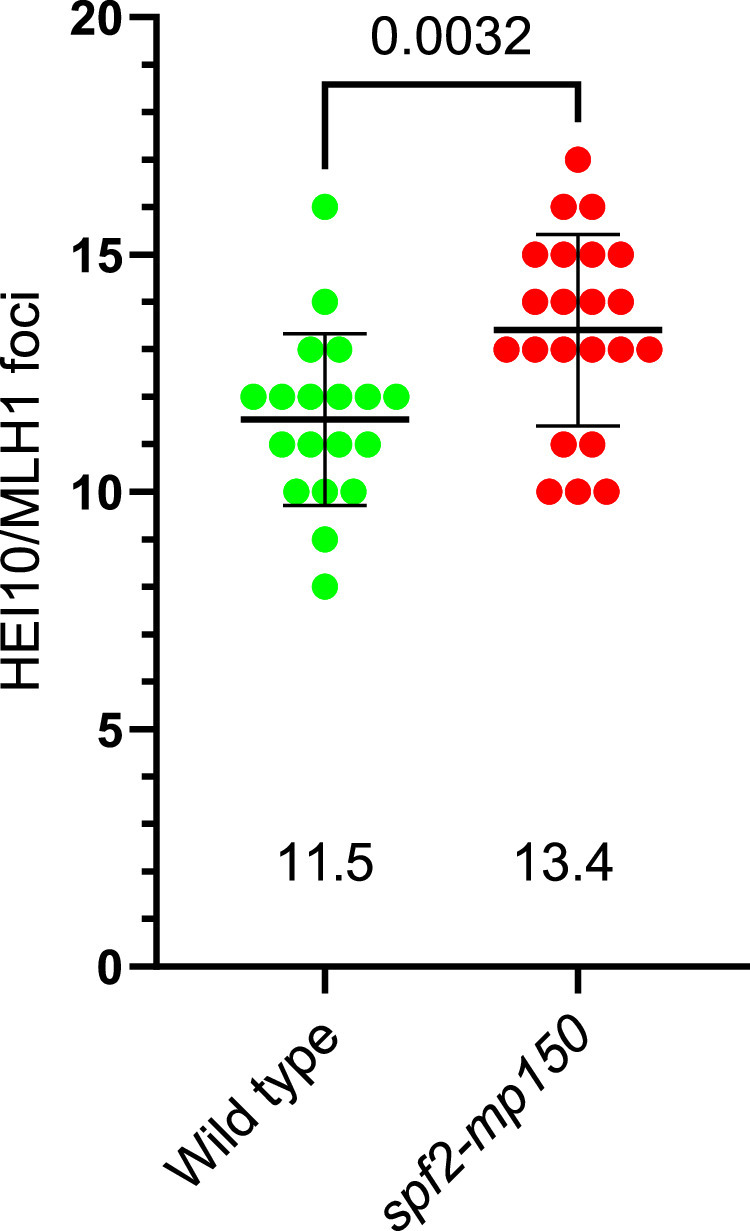
SPF2 limits class I crossovers. MLH1/HEI10 co-foci counts in diplotene-diakinesis male meiocytes in wild type and *spf2*. Each dot represents an individual cell. Black bar indicates the mean frequency and +/- standard deviation (SD). The means number of foci per cell is indicated for both genotypes and the p value is from a two-sided Mann Whitney test. Each experiment was performed at least three times. Source data is provided. Source Data

The global frequency of COs in *ctf18* and *sgo2* was similar to wild type (Mann–Whitney–Wilcoxon test, *P* > 0.1), with ~8 COs per F_2_ (Fig. 3a). Our reanalysis of available datasets for *cmt3* (*n* = 98), using the Col-CEN genome assembly, confirmed previous results with no significant difference from wild type (*P* = 0.1)^15^ (Fig. 3a and Extended Data Fig. 3). By contrast, we observed a significant increase in *spf2* with an average of 9.4 COs per F_2_ (*P* = 3 × 10^−20^ compared to the pooled wild-type plants, *P* = 0.003 compared to wild-type sister plants) (Fig. 3a and Extended Data Fig. 3). We then counted the number of HEI10/MLH1 co-foci, a marker of class I CO, which is the major CO pathway in *A. thaliana*. We observed an increase in the number of foci in *spf2* (13.4 versus 11.5, *P* = 0.003; Extended Data Fig. 4). The increase in COs measured genetically (Fig. 3a) and in the number of HEI10/MLH1 foci is of the same magnitude (+16%), suggesting that SPF2 specifically limits class I COs.

Global CO distribution along chromosomes (Fig. 3c) does not appear to be affected in *ctf18* and *sgo2* mutants, but for each of them, one pericentromeric interval had significantly more recombination than the wild type (2 Mb intervals, corrected Mann–Whitney tests, *P* = 0.022 and *P* = 0.010, respectively) (Fig. 3c). In *spf2*, COs are significantly increased in nine pericentromeric intervals and in six intervals close to chromosome ends (Fig. 3c), suggesting that SPF2 limits COs in the proximity of both centromeres and telomeres. In the *cmt3* mutant, the global CO distribution at this resolution appears largely unaffected, except in two terminal intervals where frequencies are significantly lower, consistent with previous observations^15^ (Fig. 3c).

**Extended Data Fig. 5:**
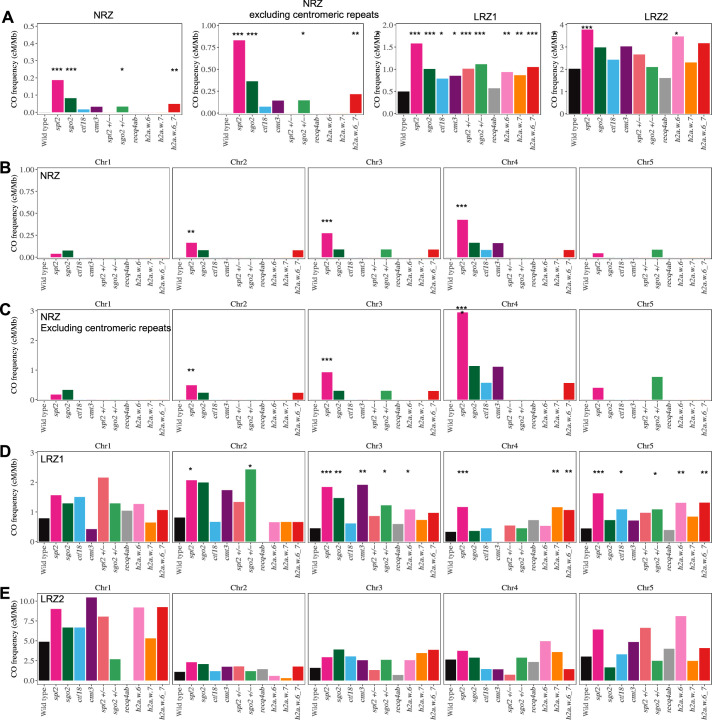
CO frequency in NRZ, LRZ1 and LRZ2 in wild type and mutants. Related to Fig. 4A. (**a**) CO frequency (cM/Mb) in NRZ, NRZ excluding the CEN178 repeat arrays, LRZ1, and LRZ2. The y-axis shows the CO frequency (cM/Mb), merging the five chromosomes. (**B-E**) Y-axis indicates the CO frequency in (**B**) NRZ, (**C**) NRZ excluding the CEN180 repeat array, (**D**) LRZ1, and (**E**) LRZ2, measured in cM/Mb for individual chromosomes. Stars correspond to two-sided Fisher exact tests with FDR correction on counts of gametes with/without a CO in the considered zone compared to wild type (* p < 0.05, ** p < 0.001, *** p < 10^−3^). The exact p values are provided in the source data. Source Data

We then examined recombination patterns close to the centromere at higher resolution (100 kb non-overlapping intervals; Fig. 4b and Supplementary Fig. 3b). In the centromeric repeat arrays (grey-shaded), no COs were detected in either wild-type (4,730 gametes) or mutant lines (2,424 gametes), suggesting that meiotic COs do not occur within the core centromere (Fig. 4b and Supplementary Fig. 3b). By definition, no COs are observed in the wild type within the NRZ, which encompasses 3.7 Mb of DNA in addition to the repeat arrays (0 CO in 4,730 gametes; see above). By contrast, and despite much smaller population sizes, we observed COs within the NRZ in *spf2* (22 COs in 724 gametes; 3.0 cM), *sgo2* (5/376; 1.3 cM), *ctf18* (1/376; 0.3 cM), *cmt3* (1/192; 0.5 cM) and *h2a.w.6 h2a.w.7* (3/380; 0.8 cM) (Fig. 4a,b, Extended Data Fig. 5, Supplementary Fig. 3b and Source data). All the observed NRZ COs are on the DNA flanking the repeat arrays (non-CEN178 NRZ) (Fig. 4b and Supplementary Fig. 3b). In the mutant with the strongest effect, *spf2*, CO density in the NRZ reached 0.2 cM Mb^−1^ if we consider the entire NRZ including the CEN178 repeats arrays and 0.83 cM Mb^−1^ if we focus on the non-CEN178-NRZ (Fig. 4a and Extended Data Fig. 5). In comparison, wild type has a CO density of 0 cM Mb^−1^ in these regions and an average of 3.1 cM Mb^−1^ genome-wide (Table 1). Unlocking of COs is observed in NRZ of each chromosome in *spf2* (Fig. 4b and Extended Data Fig. 5), and the effect is particularly marked in *spf2* in the NRZ of chromosome 4. Moving outwards in LRZ1, a significant increase in COs was also observed in *spf2*, *sgo2*, *ctf18*, *cmt3* and *h2a.w.6 h2a.w.7*, the greatest effect being observed in *spf2* (Fig. 4a,b, Extended Data Fig. 5 and Supplementary Fig. 3b). From an average of 0.5 cM Mb^−1^ in the wild-type LRZ1, CO density is increased by ~2-fold in *sgo2*, *ctf18*, *cmt3*, *h2a.w.6*, *h2a.w.7* and *h2a.w.6 h2a.w.7* and by more than 3-fold in *spf2* (Fig. 4a, Extended Data Fig. 5 and Source data). In *spf2*, the increase is significant for each chromosome and is particularly strong for chromosomes 3 and 4 (Fig. 4b and Extended Data Fig. 5). In LRZ2, CO frequency is also significantly augmented in *spf2* with an ~2-fold increase (Fig. 4 and Extended Data Fig. 5).

Although the *recq4a recq4b* mutant increases COs genome-wide to a great extent^80^, there is no significant increase in CO frequency closer to centromeres within the NRZ or LRZ regions (Fig. 4a, Extended Data Fig. 5 and Supplementary Fig. 3b). This suggests that in the absence of generic anti-CO factors like RECQ4, COs are still specifically prevented in the peri-centromeric regions.

We further investigated the CO position at the fine scale at higher resolution, using deeper sequencing of *spf2*, *ctf18* and wild-type samples containing centromere-proximal COs^81^ (median sequencing depth 9.2X, median CO resolution 1.9 kb; Source data). As expected, COs are detected in syntenic regions and are not detected in structural variants such as heterozygous inversions. COs colocalize with the low-density genes present in the NRZ and LRZ1, even when genes are adjacent to the centromere repeat arrays (for example, left of the centromere on chromosomes 3 and 5) (Fig. 4c and Supplementary Figs. 4 and 5), consistent with previous reports that COs tend to happen in genes^8,45^.

### *spf2* and *sgo2* have a semi-dominant effect on pericentromeric COs

**Fig. 5:**
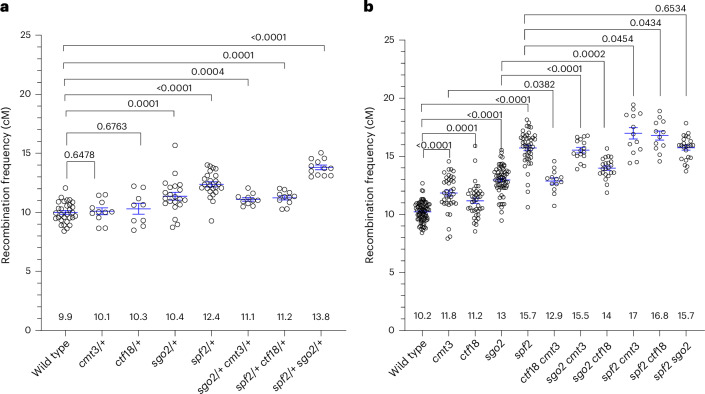
Cumulative effect of mutations on frequency of centromere-proximal CO. Recombination (cM) measurement in CTL.3.437.55, as in Fig. 1a,b. **a**, Heterozygous mutants *ctf18/+*, *cmt3/+*, *sgo2/+*, *spf2/+* and combinations. *P* values are two-sided Mann–Whitney tests (*n* = 30, 11, 9, 21, 24, 10, 11 and 12, respectively). **b**, Single and double homozygous mutants. These figures pool the data from several populations, and the data for individual experiments are shown in Extended Data Figs. 7 and 8. Blue lines indicate the mean of the recombination frequency ± s.e.m., and its value (cM) is indicated along the *x* axis for each genotype. *P* values are two-sided Mann–Whitney tests (*n* = 81, 37, 38, 63, 44, 13, 16, 21, 13, 12 and 23, respectively). Mutant alleles are *cmt3-11*, *ctf18-mp193*, *sgo2-1* and *spf2-3*, all in the Col background. Source data

Dominant mutation could facilitate the manipulation of CO rates in plant breeding schemes. We tested the effect of heterozygous mutation of *SPF2*, *CTF18*, *CMT3* and *SGO2* on pericentromeric COs. In the CTL3.437.55 interval, we found a significant CO increase in *spf2/+* and *sgo2/+* but not in *cmt3/+* and *ctf18 /+* (Fig. 5a and Extended Data Fig. 7). We then analysed genome-wide recombination in hybrids *spf2/+* and *sgo2*/+ using progeny sequencing, as described above. The average CO numbers per genome were similar to wild type (Fig. 3a). In the NRZ, we did not detect COs in *spf2/+* (*n* = 187 F_2_ plants) and found one in *sgo2/+* (*n* = 188 F_2_ plants) (Fig. 4a and Extended Data Fig. 5). In LRZ1, CO events were ~2-fold more frequent than in the wild type in both *spf2*/+ and *sgo2*/+ (Fig. 4a, Extended Data Fig. 5 and Supplementary Figs. 3 and 5). The *spf2* and *sgo2* mutations are thus semi-dominant, the heterozygous mutant having more proximal CO than wild type but less than in the homozygous mutants.

### Parallel pathways repress COs in pericentromeres

Single mutations in *CTF18*, *SPF2*, *SGO2* and *CMT3* increase centromere-proximal COs; we aimed to test whether they could have a cumulative effect, which would suggest that they belong to parallel pathways of CO repression. We measure CO frequencies in the CTL3.437.55 interval in the six possible double mutants, together with single mutants and wild-type plants from the same segregating populations (Fig. 5b and Extended Data Fig. 8). We confirmed an increase of COs in each single mutant, compared to the wild type, with *spf2* showing the most pronounced effect (Fig. 5b and Extended Data Fig. 8).

All six double mutants had increased COs compared to the wild type (Fig. 5b and Extended Data Fig. 8). To determine whether the effect on recombination was cumulative, we compared each double mutant to the highest single mutant in its respective combination. The double mutants with *cmt3*—*ctf18 cmt3*, *sgo2 cmt3* and *spf2 cmt3*—showed significantly higher recombination than the corresponding single mutants (Fig. 5b and Extended Data Fig. 8). This suggests that CMT3 prevents centromere-proximal CO formation through parallel mechanisms relative to SPF2, CTF18 and SGO2. Similarly, *spf2 ctf18* and *sgo2 ctf18* had significantly higher recombination than *spf2* and *sgo2*, suggesting that CTF18 suppresses COs independently from SPF2 and SGO2. However, *spf2 sgo2* recombination was not increased compared to *spf2*, the highest single mutant, implying that SPF2 and SGO2 may operate through a common pathway to prevent centromere-proximal COs (Fig. 5b and Extended Data Fig. 8). The two double mutants *spf2 cmt3* and *spf2 ctf18* had the highest recombination rate in CTL3.437.55 observed so far, an increase of ~60% compared to the wild type.

### Enhanced pericentromeric CO and genome stability

**Extended Data Fig. 6:**
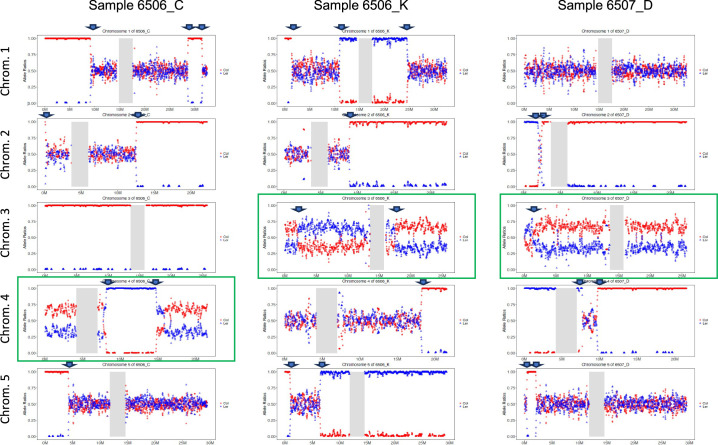
CO analysis in trisomic samples. The plots show the Col/L*er* allele frequency (Red/Blue) along the five chromosomes on the three aneuploid samples detected in the *spf2* progeny. Sample 6506_C is trisomic for chromosome 4, 6506_K and 6507_D are trisomic for chromosomes 3, as shown by higher sequence coverage and 2:1 allelic ratio. CO sites are indicated by arrows.

Previous research suggested that centromere-proximal COs can cause chromosome mis-segregation at meiosis, notably in humans^31,32^. We screened for aneuploid by analysing the sequences of *ctf18*, *sgo2* and *spf2* mutant progenies (F_2_ populations from Col/L*er* hybrids), using unbalanced sequence coverage between chromosomes as the detection criteria^81–83^. In wild type (*n* = 2,365), *ctf18* (*n* = 188), *cmt3* (*n* = 96), *sgo2* (*n* = 188), *sgo2*+/− (*n* = 188) and *spf2*+/− (*n* = 187), we did not detect any aneuploids, suggesting that increasing COs at the centromere-proximal regions in these mutants does not lead to higher aneuploidy rates. However, we found three trisomic plants in the progeny of *spf2* (0.8%, *n* = 365), suggesting a slight genomic instability. The three trisomies were not associated with the detection of a CO in the NRZ/LRZ1 (Extended Data Fig. 6). However, we cannot exclude the possibility that a proximal CO was present in the meiotic cell at the origin of the trisomies and caused the mis-segregation, but is not detectable in the trisomics. Indeed, a CO is a reciprocal exchange between two of the four chromatids of a bivalent. In case of mis-segregation resulting in the presence of two chromatids in a gamete and trisomy in the offspring, the CO would be detected if one of two chromatids was recombined and the other not. If the gamete inherited the two non-recombinant chromatids or the two reciprocal recombinant chromatids, the CO would not be detectable by sequencing. Alternatively, the chromosome mis-segregation events may not be the consequence of a proximal CO but rather an independent effect of the *spf2* mutation on cohesion and/or monopolar orientation^58^.

**Fig. 6:**
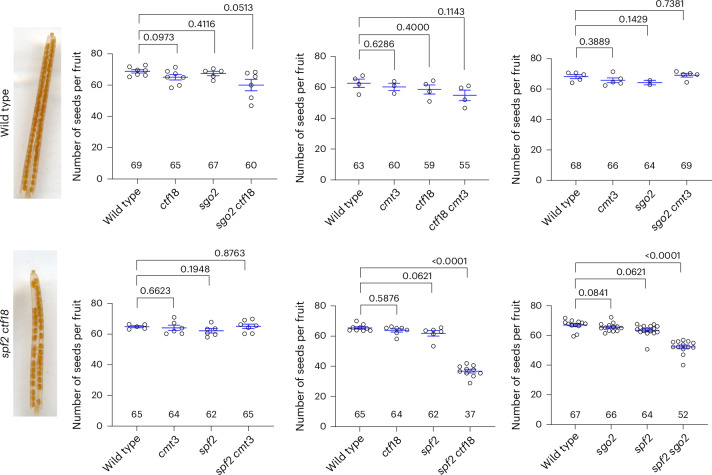
Fertility in mutants with elevated centromere-proximal COs. Fertility was assessed by counting the number of seeds per fruit in 10 fruits on a minimum of three plants per genotype. Each dot represents the average number of seeds per fruit for an individual plant. Blue lines indicate the mean ± s.e.m., and its value is indicated along the *x* axis. Representative pictures of wild-type and *spf2 ctf18* fruits are shown. Statistical comparisons are two-sided Mann–Whitney tests (*n* = 4, 3, 4, 4, 7, 7, 5, 6, 5, 5, 2, 5, 5, 6, 6, 7, 9, 7, 6, 10, 12, 13, 16 and 13, respectively). Mutant alleles are *cmt3-11*, *ctf18-mp193*, *sgo2-1* and *spf2-3*, all in the Col background. Source data

Next, we assessed the fertility of mutants with elevated pericentromeric CO, as measured by the average number of seeds per fruit (Fig. 6). None of the *cmt3*, *ctf18*, *sgo2* and *spf2* single mutants showed a reduction in fertility compared to their respective sister wild-type plants. Similarly, in the double mutants *ctf18 cmt3*, *sgo2 cmt3*, *spf2 cmt3* and *sgo2 ctf18*, in which proximal COs are further elevated, the fertility was not significantly reduced compared to the wild type. However, fertility was reduced by 43% in *spf2 ctf18* and by 22% in *spf2 sgo2* compared to their sister wild-type plants (Fig. 6). In *spf2 ctf18*, we observed a mild meiotic defect, with infrequent precocious separation of sister chromatids and segregation defect at meiosis II (Extended Data Fig. 9 and Supplementary Fig. 6). In *spf2 sgo2*, we could not detect a clear meiotic defect (Extended Data Fig. 10 and Supplementary Fig. 7). The observed reduction in fertility does not correlate well with increased proximal recombination measured in CTL3.347.55. Combining *spf2* and *sgo2* mutation does not increase COs in this interval compared to the single mutants but does reduce fertility (Figs. 5b and 6). The highest levels of proximal COs were observed in *spf2 cmt3* and *spf2 ctf18* (Fig. 5b), but fertility is affected only in *spf2 ctf18* (Fig. 6).

## Discussion

The phenomenon of CO suppression near centromeres is evolutionarily conserved and is an important limitation for plant breeding. In *A. thaliana*, complete suppression of COs spans the 12 Mb centromeric repeat arrays and an additional 4 Mb of flanking chromatin. Furthermore, an additional 10 Mb flanking region (LRZ1) shows a severe reduction in COs (~8-fold less CO per Mb compared to chromosome arms).

This study shows that CTF18 and SGO2 play a role in restricting COs near centromeres in *A. thaliana*, both being regulators of cohesin in eukaryotes. CTF18 is a replication factor that participates in sister chromatid cohesion establishment^84^. Its role is conserved in *A. thaliana* but is not essential, making the growth and development of the *ctf18* single mutant indistinguishable from wild type^71^ (Supplementary Fig. 2). SGO family proteins protect centromeric cohesion during meiosis I in eukaryotes^72,85^, a function that is supported in *A. thaliana* by both SGO1 and SGO2, with SGO1 playing a prominent role^73,74^. SGO1 and SGO2 also promote monopolar orientation of the kinetochore, with SGO2 playing a prominent role^58^. While SGO2 had an established role at metaphase/anaphase I, it also localizes at pericentromeres throughout prophase (Supplementary Fig. 2). Our result thus suggests that proximal COs are prevented by cohesins, and lessening cohesin loading or protection is sufficient to permit proximal COs. It should be noted that cohesins appear to have opposite roles in regulation of CO formation, as cohesins are also pro-CO factors, as shown by the failure of meiotic DSB repair in *rec8* and *scc3-1* mutants^86^. Further, we showed that a specific mutation of an essential cohesin subunit, SMC1, leads to a redistribution of CO to more distal regions of chromosomes. These apparently opposite functions of cohesins likely reflect the multiple roles of cohesins in genome organization, supporting both cohesion of replicated chromatid and chromatin folding through loop formation^87^. In both budding and fission yeast, cohesin regulation also contributes to CO suppression in centromere-proximal regions^2,52^. The REC8 cohesin was reported to show centromere proximal enrichment in *A. thaliana*^88^, and natural genetic variation in *REC8* was associated with CO patterning in barley^89^. The enrichment of cohesins at the centromeres could lead to CO suppression by modifying how chromatin loops are folded and/or by regulating several steps of the meiotic recombination pathway: (1) A high cohesin density may prevent DNA DSB formation by limiting access to SPO11 and associated factors^2^. (2) Cohesin enrichment near centromeres may promote sister chromatid-mediated DSB repair, thereby repressing COs. Studies in budding yeast support this model, showing that cohesins influence template choice for DSB repair during vegetative growth^53^. (3) Cohesins may favour the repair of recombination intermediates into non-COs rather than COs, as suggested by a higher non-COs/CO ratio in the vicinity to centromeres in humans^26^.

We show that disruption of *SPF2* leads to a large increase in CO formation in both the NRZ and the LRZs. This increase is larger than in any of the *A. thaliana* mutants described so far. SPF2 is a homologue of the yeast deSUMOylase Ulp2. Ulp2 regulates cohesins and condensins turnover by preventing the assembly of SUMO chains on structural maintenance of chromosomes (SMC) complexes^75^. Thus, one possible function of SPF2 is the regulation of cohesins near centromeres, similar to CTF18 and *SGO2*. This hypothesis is supported by several observations: (1) in combination with other mutations, *spf2* leads to premature loss of sister chromatid cohesion at meiosis^58^, (2) the amount of REC8 cohesin is reduced on metaphase meiotic chromosomes in *spf2*^58^ and (3) combining *spf2* with *sgo2* did not lead to further CO increase in the proximal interval tested, suggesting that they act in the same pathway of CO suppression. SPF2 may directly regulate the activity of SGO2 in cohesion protection, a hypothesis supported by the known regulation of SGO turnover by SUMO in yeast^90^. However, the observation that the mutation of *SPF2* leads to a higher frequency of proximal CO than *sgo2* and the reduced fertility in *spf2 sgo2* suggests that SPF2 has additional targets. The *spf2 ctf18* double mutant has more proximal COs than the single mutants, suggesting that they act via parallel pathways. One possibility is that they cumulatively regulate cohesins, which is suggested by the reduced fertility and meiotic defects of the double mutant. Alternatively, SPF2 may contribute to CO suppression independently of cohesins, possibly targeting kinetochores or centromeric chromatin^52,91,92^. Lastly, SPF2 may also directly regulate the SUMOylation of the pro-CO recombination machinery, such as HEI10 and MSH4, which were shown to be SUMOylated in yeast^93,94^. Further investigation is required to test these hypotheses regarding the mechanism by which SPF2 limits proximal COs.

The DNA methylase CMT3 contributes to proximal CO suppression^15^. Our data showed that *cmt3* has cumulative effects on recombination near centromeres (CTL3.437.55) with *sgo2*, *ctf18* and *spf2*, indicating that DNA methylation and cohesin regulation independently contribute to centromeric CO suppression. One possible scenario is that DNA methylation promotes closed chromatin and that cohesin acts at a different scale promoting folding of chromatin fibres and/or interacting with the recombination machinery.

A mutation in the *A. thaliana CENPC* reduces COs in a tested centromere-proximal interval (Fig. 1b). This is in contrast to budding yeast^52^, in which mutating kinetochore genes increases COs close to centromeres. However, it should be noted that we tested only one specific hypomorphic mutation in the *CENPC* essential gene, and further work testing additional mutations in kinetochore components would be required to conclude on a potential role on recombination in *A. thaliana*.

In *Drosophila*, humans and yeasts, centromere-proximal COs were associated with chromosome segregation defects^2,31,32,34^. Despite the observed increase in centromere-proximal COs, fertility remains unaffected in *spf2*, *ctf18* and *sgo2* mutants. Further, we did not detect any aneuploids in the progeny of *ctf18* and *sgo2* mutants and detected very few trisomic plants in the progeny of *spf2* (0.8%). This suggests that enhancing centromere-proximal COs is possible, at least to a certain extent, without drastically impacting chromosome segregation.

The combined challenges of food security and sustainable agriculture call for efficient plant breeding, which relies on meiotic recombination. Mutating generic anti-CO factors was shown to increase COs in model and crop species, but the effect is limited to chromosome arms and does not unlock pericentromeric regions that remain inaccessible to breeding^78,79,95–99^. The association observed in barley between natural genetic variation in the REC8 cohesin gene and CO patterning suggests that the role of cohesin regulators in suppressing proximal COs might be conserved in cereal crops^89^. In this study, *SPF2*, *CTF18* and *SGO2* emerged as promising targets for increasing centromere-proximal COs in plants. The semi-dominant effect observed in *spf2*+/− and *sgo2*+/− may facilitate their usage in breeding schemes. It should be noted that suggested usage in breeding of these mutations does not imply that the mutations will be present in the commercial varieties. Indeed, increasing CO in pericentromeric regions would be useful to introgress traits from non-elite source (for example, related wild species) into elite varieties. This occurs in the early phase of a breeding scheme, and the mutation(s), after having allowed the desired recombination events, can be segregated out together with undesired genomic regions from the source and thus not be present in the final varieties. In this context, the usage of a mutation in breeding would not be hindered by a reasonable penalty on growth or fertility. It would be interesting to test whether the observation made in *A. thaliana* can be transferred to species such as tomato and cereals, in which very large pericentromeric regions are devoid of COs^4,5,23,40^.

## Methods

### Plant materials and growth conditions

*A. thaliana* plants were grown in a greenhouse or growth chamber under controlled long-day conditions (16 h day/8 h night, 20 °C). The *A. thaliana* lines used in this study were as follows: fluorescent lines to measure recombination: CG437 (CS71169) and CR55 (ref. ^58^) (CS71173)^56^; mutant lines used in the study were as follows: *cenpc* (*cenpc-mp221*) Col-0 (ref. ^58^); *ctf18* (*ctf18-mp193/ctf18-4*) Col-0 (ref. ^58^); *ctf18* (*ctf18-5*, this study, Supplementary Fig. 1) L*er*; *smc1* (*smc1-mp172*) Col-0 (ref. ^58^); *smc3* (*smc3-mp226*) Col-0; *zyp1* (*zyp1-1*) Col-0 and *zyp1* (*zyp1-6*) L*er* (ref. ^68^); *cmt3* (*cmt3-11*-SALK_148381) Col-0 (ref. ^15^); *smc1* (FLAG024C06, line DPF3) Wassilewskija^100^; *sgo2* (*sgo2-1* line 34303) Col-0 (ref. ^73^); *sgo2* (*sgo2-2*, this study, Supplementary Fig. 1) L*er*; *spf2* (*spf2-3* SALK_140824) Col-0 (ref. ^101^); *spf2* (*spf2-6*, this study, Supplementary Fig. 1) L*er*. Plants were genotyped by PCR with an annealing temperature of 58 °C (primers in Supplementary Table 1).

### Recombination measurements using FTLs

To measure recombination, we utilized FTLs developed in the Col-0 accession^56^. We crossed a plant carrying the transfer DNA (T-DNA) line CG437 (CS71169) with a GFP marker to another plant carrying the T-DNA line CR55 (CS71173) with a dsRed fluorescent protein marker. Both fluorescent proteins are expressed in seeds under the control of the *NapA* promoter. These T-DNA markers flank the centromere on chromosome 3. We obtained F_1_ plants that displayed both red and green fluorescence in *trans*. Our goal was to have these colours in *cis* configuration to measure recombination between the two markers. To achieve this, we grew the F_2_ progeny and identified plants that were homozygous for both the red and green T-DNA markers, indicating that the markers were in *cis* configuration. We also selected plants that were homozygous for one marker and heterozygous for the other, as they also had the markers in *cis* on one chromatid. These selected plants were used in further crosses with candidate genes to measure recombination.

We then crossed the candidate gene with the *cis* configuration line, named CTL3.437.55, to obtain F_1_ seeds. These seeds were grown into plants heterozygous for both the candidate gene and the red and green T-DNA markers. These plants were self-pollinated to obtain F_2_ seeds. From the F_2_ seeds, we preselected those that were heterozygous for both T-DNA markers, as heterozygosity is required to visualize recombination events. We then genotyped the F_2_ plants to identify those that were homozygous mutants for the candidate gene, as well as their wild-type siblings. We allowed these plants to self-pollinate to produce F_3_ seeds, in which recombination was measured for both the homozygous mutant and sister wild-type plants.

**Extended Data Fig. 7:**
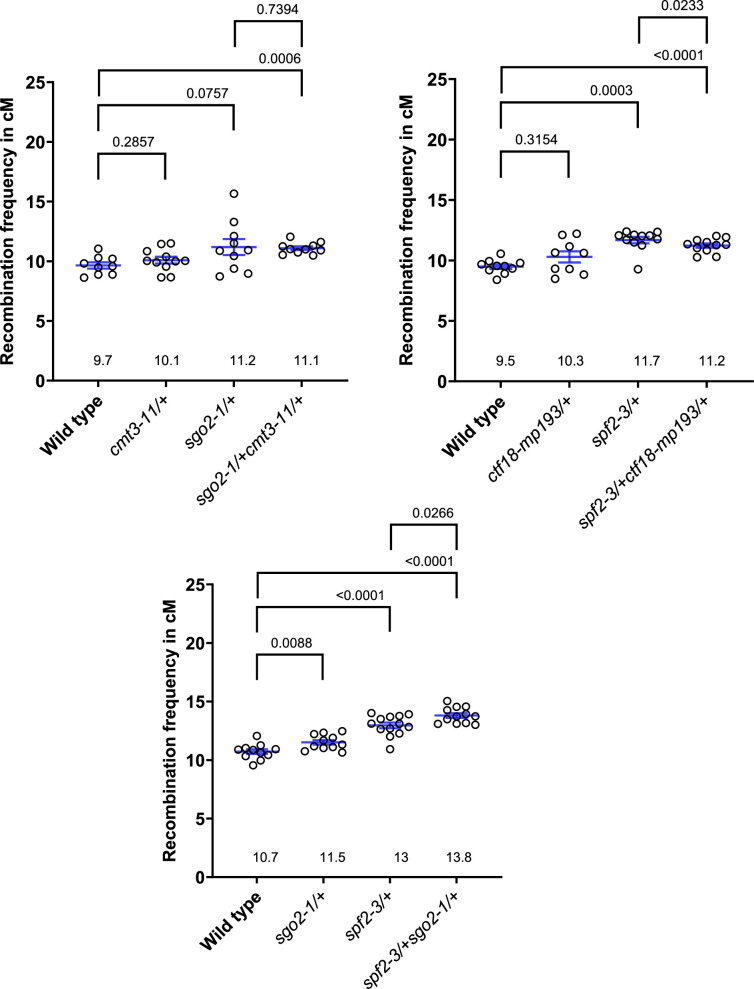
Measurement of recombination in CTL.3.437.55 in individual populations of heterozygous mutants. Related to Fig. 5a. Measure of recombination frequency in centimorgans (cM) in the CTL.3.437.55 interval. Each dot represents a measurement on ~500 seeds. Blue lines indicate the Mean of the recombination frequency +/- standard Error (SEM) and the value (cM) is given for each genotype. Single heterozygous, double heterozygous mutants and wild types from the same segregating population (sister plants) are compared. Statistical tests are two-sided Mann-Whitney tests. Source data is provided. Source Data

**Extended Data Fig. 8:**
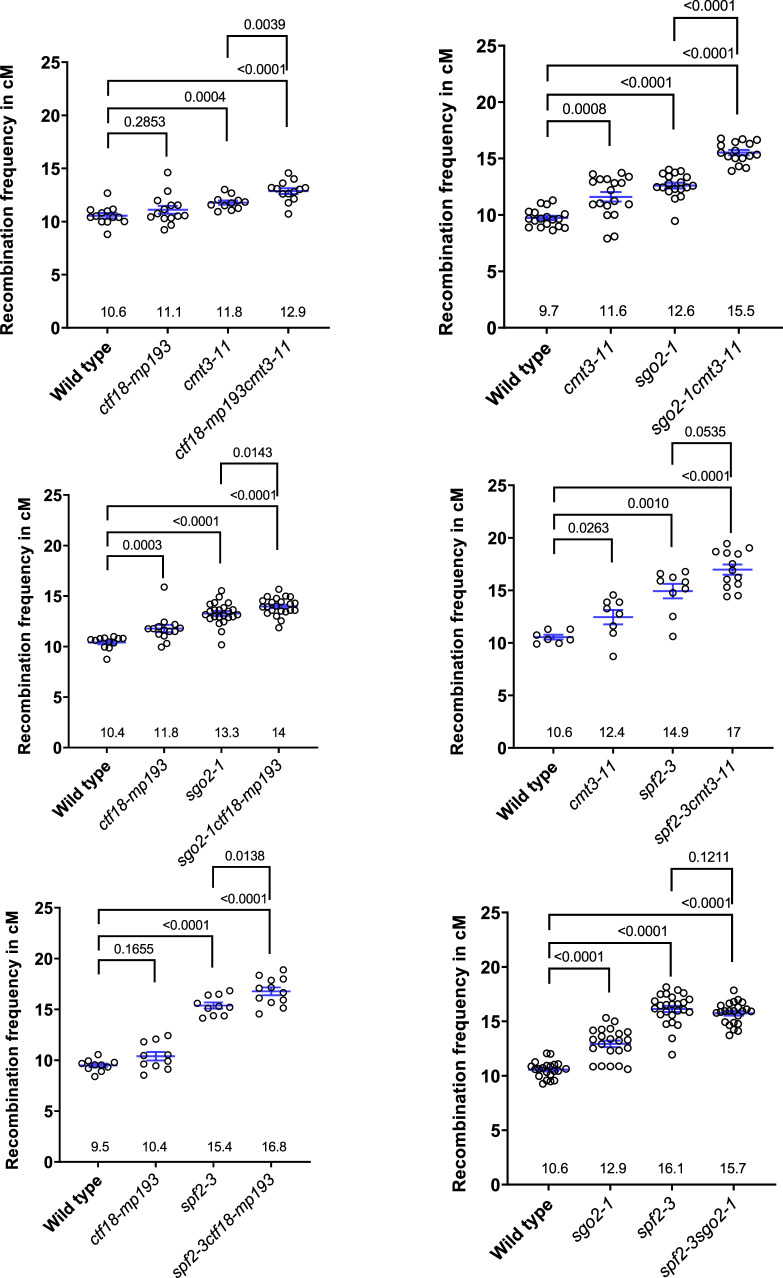
Measurement of recombination in CTL.3.437.55 in individual populations of double mutants. Related to Fig. 5b. Measure of recombination frequency in centimorgans (cM) in the CTL.3.437.55 interval. Each dot represents a measurement on ~500 seeds. Blue lines indicate the Mean of the recombination frequency +/- standard Error (SEM) and the value (cM) is given for each genotype. Each double-mutant mutant is compared to single mutants and wild types from the same segregating population (sister plants). Statistical tests are two-sided Mann-Whitney tests. Source data is provided. Source Data

**Extended Data Fig. 9:**
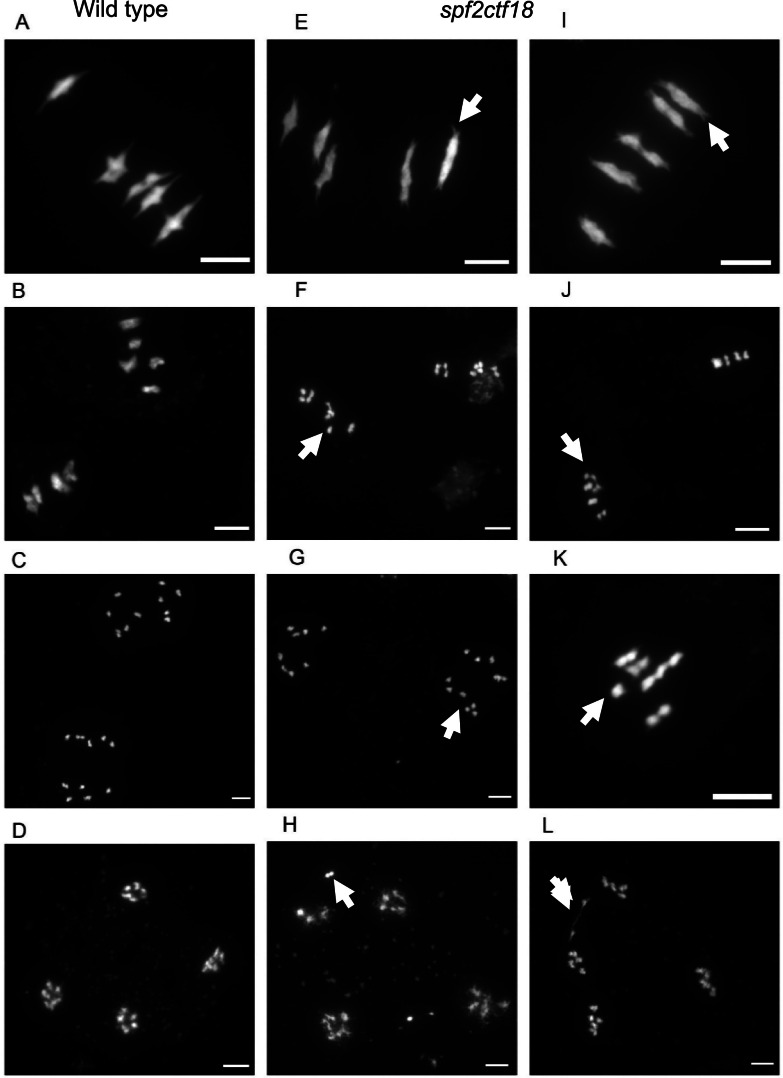
Meiotic Chromosome spreads in wild type and *spf2 ctf18*. Meiotic chromosome spreads were visualized using DAPI (4′,6-diamidino-2-phenylindole) staining in wild type (panels **A–D**) and *spf2 ctf18* double mutants (panels **E–L**). Representative spreads are shown for metaphase I (**A, E, I**), metaphase II (**B, F, J, K**), anaphase II (**C, G**), and telophase II (**D, H, L**). In panel **K**, a single metaphase II plate is highlighted. White arrowheads indicate meiotic defects, including kinetochore splitting at metaphase I and chromatid splitting, chromatin bridges, lagging chromosomes or unequal chromatid segregation during meiosis II. The quantification of these defects is presented in Supplementary Fig. 6. Scale bar: 5 µm. Mutant alleles are *ctf18-mp193* and *spf2-3*.

**Extended Data Fig. 10:**
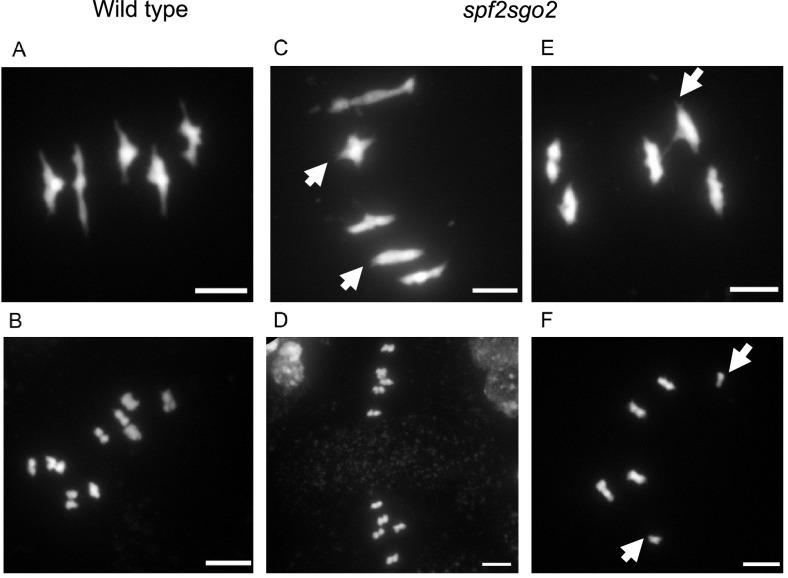
Meiotic Chromosome spreads in wild type and *spf2 sgo2*. Meiotic chromosome spreads were visualized using DAPI (4′,6-diamidino-2-phenylindole) staining in wild type (panels **A–B**) and *spf2 ctf18* double mutants (panels **C–F**). Representative spreads are shown for metaphase I (**A, C, E**), metaphase II (**B, D, F**). In panel **F**, a single metaphase II plate is highlighted. White arrowheads indicate meiotic defects, including kinetochore splitting at metaphase I and chromatid splitting during meiosis II. The quantification of these defects is presented in Supplementary Fig. 7. Scale bar: 5 µm. Mutant alleles are *sgo2-1* and *spf2-3*.

For recombination analysis, we used two different methods: one using CellProfiler 2.1.1, a previously established method^102^, and another established in the group using Fiji v1.53c image analysis software, coupled with a pipeline for data analysis using Perl 5.34.0 and R 1.3.1093 described in ref. ^103^. Seed preparation differed between the two methods. For CellProfiler, seeds were spread evenly on a black surface using a microscopic slide to prevent overlap. For the Fiji method, seeds were placed on 1% agar in small Petri dishes to ensure separation and prevent overlap. For Fig. 1b and Extended Data Fig. 1c–g, recombination frequency was measured using the Fiji image analysis software method and by placing seeds on 1% agar. For Fig. 5, Extended Data Fig. 7 and Extended Data Fig. 8, recombination frequency was measured using the CellProfiler method and by using dry seeds.

In both methods, three images were acquired for each sample: (1) brightfield, (2) UV through a dsRed filter and (3) UV through a GFP filter, using a fluorescent stereomicroscope. CellProfiler/Fiji image analysis (version 2.1.1) was used to identify seed boundaries in the micrograph images and assign fluorescence intensity values for RFP and GFP to each seed. Genetic distance was calculated using the following formula:

$$c{\rm{M}}=100\times (1-[1-2({N}_{{\rm{G}}}+{N}_{{\rm{R}}})/{N}_{{\rm{T}}}]1/2)$$

where *N*_G_ is the number of green-alone fluorescent seeds, *N*_R_ is the number of red-alone fluorescent seeds and *N*_T_ is the total number of seeds counted^102^. The Mann–Whitney test was used for statistical comparison between samples.

### CRISPR–Cas9 mutagenesis of *AtSGO2, AtCTF18* and *AtSPF2*

All guide RNAs were designed based on the *A. thaliana* Columbia-0 genome and verified for specificity against the Landsberg genome using the CRISPR-P 2.0 tool http://crispr.hzau.edu.cn/CRISPR2/ with the U6 promoter. Two guides were selected at both the 3′ and 5′ ends, excluding untranslated regions (Supplementary Table 1 and Supplementary Fig. 1). The guides, rich in GC content, were 19 bp long, lacked repeats and had the PAM sequence in the reverse complementary orientation. The guides were cloned into the plCH86966 plasmid using CloneAmp at 65 °C. In the first reaction, each guide (in total four guides per gene) was assembled into a vector that finally comprises the AtU6 promoter and guide RNA expression cassette. The vector was transformed into *Escherichia coli*, purified and Sanger-sequenced to confirm key features (restriction sites, the anchor site of each plasmid (reverse complementary) and the guide). Cloning of the guide RNAs into the final Cas9-containing binary vector was carried out using the MoClo Golden Gate cloning toolkit^104^. In the second reaction, all four plasmids containing guide RNA were subcloned into the final binary vector, assembled with the intron-containing Cas9 (ref. ^105^) expression cassette, Fast-Red expression cassette (that allowed selection of Cas9 transformants by selecting for fluorescent red seeds) and linker, transformed into competent cells, selected with kanamycin and verified by restriction digestion. The complete plasmid structure was confirmed by Nanopore sequencing (https://www.plasmidsaurus.com/), and the plasmids were transformed into *Agrobacterium* (GV3101), selected with appropriate antibiotics and verified by PCR. Transformed bacteria were selected from an overnight preculture in LB media containing gentamycin, kanamycin and rifampicin. These were then incubated in fresh culture for approximately 5 h until reaching an optical density of 0.5–0.6. Bacteria were centrifuged (4,000 *g* for 10 min), resuspended in a freshly prepared 5% sucrose solution with 20 μl of Silwet L-77 and used to dip young flower buds of about 16 Columbia and Landsberg plants per batch for 15 s. The plants were then covered with dark plastic bags overnight. Transformed seeds (T_1_) were selected based on red fluorescence; Cas9 amplification by PCR was performed on T_1_ plants, and mutations confirmed by PCR and by Sanger sequencing. T_1_ plants were selfed, and in the T_2_ generation, we selected for mutants that are Cas9-free both based on selecting non-fluorescent seeds and validating the T_2_ plants by PCR, genotyping for the mutation in the gene and validating via sequencing. If the T_2_ plant is homozygous for mutation, then the plant was backcrossed to L*er* before crossing it to *sgo2-1*, *ctf18-mp193* and *spf2-3* alleles in Col-0. If the T2 L*er* plant was with heterozygous mutation, then the plant was directly crossed to its allele in Col-0. This way we produced *ctf18-mp193/ctf18-del* at G181, *spf2-3/spf2-del* at L90 and *sgo2-1/sgo2-del* at K74 Col/L*er* F_1_ hybrids. These F_1_ plants were validated by PCR for the mutations both in Col and in L*er* background and were selfed to produce F_2_ progeny, which were sequenced (see below) to obtain genome-wide CO landscape.

### Genome-wide sequencing

From the set of 334,680 high-quality Col/L*er* single-nucleotide polymorphisms (SNPs) created from a previous study^8^, we further filtered those SNPs that distributed outside the 0.05-quantile and 0.95-quantile of total read number, Col allelic ratio (Col read number divided by total read number) and L*er* allelic ratio (L*er* read number divided by total read number), homozygous Col allelic ratio (number of samples with 0/0 genotype divided by the total number of genotyped samples), heterozygous Col allelic ratio (number of samples with 0/1 genotype divided by total number of genotyped samples), homozygous L*er* allelic ratio (number of samples with 1/1 genotype divided by total number of genotyped samples) and the total number of genotyped samples. Finally, a set of 234,744 golden SNPs (provided as Supplementary Data 1) was kept for subsequent analysis.

Leaf samples from F_2_ populations were used for DNA purification and library preparation as described in ref. ^77^ for Illumina sequencing (NextSeq 2000 2 × 150 bp), performed by the Max Planck-Genome-Centre (https://mpgc.mpipz.mpg.de/home/). We generated and sequenced F_2_ plants from Col/L*er* F_1_ plants selected in a population segregating for *spf2* (number of sequenced F_2_ plants: derived from F_1_ wild type, *n* = 284; from *spf2*+/−, *n* = 187; and from *spf2*−/−, *n* = 365), *ctf18* (wild type, *n* = 188 and *ctf18*−/−, *n* = 188) or *sgo2* (*sgo2*+/−, *n* = 188 and *sgo2*−/−, *n* = 188). The F_2_ sequence dataset for *cmt3* (wild type, *n* = 350 and *cmt3*−/−, *n* = 96) and *h2a.w.6_7* (*h2a.w.6*, *n* = 191; *h2a.w.7*, *n* = 189; and *h2a.w.6 h2a.w.7*, *n* = 190) was downloaded from previous studies^15,45^. The sequence dataset of the backcross population *recq4a recq4b* (Col/L*er* F_1_ *recq4a*−/− *recq4b*−/− crossed to wild-type Col, as male or as female, *n* = 233 gametes) was downloaded from a previous study^80^. We also included F_2_ wild-type samples from ref. ^77^. All these data were analysed/reanalysed with the genome assembly Col-CEN v1.2. (ref. ^3^). The golden SNPs were genotyped by using inGAP-family^106^, and then a sliding window-based method was used for predicting COs, using a window size of 50 kb and a step size of 25 kb^81,82,106^. We further filter false close double COs that commonly present across samples, which are caused by mis-genotyping.

For high-resolution mapping, we selected samples with centromere-proximal COs and re-sequenced with high depths, including samples from the *spf2* population (wild type, *n* = 23; *spf2*+/−, *n* = 18; *spf2*−/−, *n* = 101) and the *ctf18* population (wild type, *n* = 11; *ctf18*−/−, *n* = 20). We also included proximal COs from wild-type F_2_, male and female BC1 from previous study^8,77,81^. We manually examined centromere-proximal COs with a high resolution.

The NRZs were defined as the contiguous regions containing the main CEN178 arrays where COs are completely absent in the wild type. The external borders of the NRZ are thus defined by the closest CO to the centromere detected in the wild type (19,167 COs from 4,730 gametes). Moving outwards, we defined the LRZ1 as spanning 0.5 cM on each side flanking the boundaries of NRZ (defined by the position of the 24th CO on each side counting from the centromere), and LRZ2 as spanning 0.5 cM on each side flanking the LRZ1 (defined by the position of the 47th CO on each side counting from the centromere) (Fig. 3b).

### Chromosome spreads preparations

Chromosome spreads were prepared as described previously^107^. Young inflorescences were collected from *A. thaliana* plants when they were 5–6 weeks old, and inflorescences were fixed in Carnoy’s fixative (3:1 absolute ethanol/glacial acetic acid). The fixative was refreshed until the solution was clear. The buds in fixative can be stored at 4 °C. Flower buds 0.5–0.6 mm in size were isolated from inflorescences and washed in 10 mM citrate buffer at pH 4.5 then incubated in digestion mix (0.3% (*w*/*v*) Pectolyase Y-23 (MP Biomedicals), 0.3% (*w*/*v*) Driselase (Sigma) and 0.3% (*w*/*v*) Cellulase Onozuka R10 (Duchefa) for 2–3 h at 37 °C. Two to three digested buds were transferred to a clean slide and transformed into a fine cell suspension with a bent dissection needle. Then 10 µl of 60% glacial acetic acid was added to the mixture and stirred gently at 45 °C on a hotplate for 2 min. After 1 min, one more 10 µl drop of 60% glacial acetic acid was added to the slide and the sample stirred gently. Stirring likely facilitates in removing the cytoplasm as much as possible. Cells were fixed on the slide by adding ice-cold 3:1 Carnoy’s fixative first around the droplet and then directly flushed on the drop. Excess of fixative is removed by tilting the slide onto the beaker. Slides were left to dry tilted at room temperature, then 12 µl of DAPI in anti-fade mounting media (Vectashield) was applied to the slide, and a coverslip was added. Chromosomes were visualized using a Zeiss Axio Observer epifluorescence microscope.

### Immunolocalization

Chromosome spreads and immunolocalization was performed on meiotic cells with preserved three-dimensional structure^68^. Flower buds, 0.5–0.6 mm, were dissected to expose young anthers and were collected in buffer A (80 mM KCl, 20 mM NaCl, 15 mM Pipes–NaOH, 0.5 mM EGTA, 2 mM EDTA, 80 mM sorbitol, 1 mM DTT, 0.15 mM spermine and 0.5 mM spermidine). Buds were fixed in buffer A containing 2% paraformaldehyde for 30 min under vacuum, washed twice for 5 min in buffer A and digested at 37 °C for 60 min in citrate buffer containing 0.3% cellulase, 0.3% pectolyase Y23, 0.3% driselase and 0.1% sodium azide. After digestion, the buds were washed twice in buffer A, and finally buds were kept on ice in buffer A.

For embedding, 10 buds were placed in 6 µl buffer A on an 18 × 18 mm coverslip, and anthers were dissected and squashed to release meiocytes. A 3 µl drop of activated polyacrylamide solution (15% polyacrylamide with sodium sulfite and ammonium persulfate) was added, a second coverslip was placed on top and gels were allowed to polymerize for 1 h before separating the coverslips. The cover slip containing the gel pad was incubated in 1× PBS containing 1% Triton X-100 and 1 mM EDTA for 1 h, followed by blocking for 2 h in 3% bovine serum albumin in 1× PBS with 0.1% Tween-20.

Samples were incubated with primary antibodies in blocking buffer at 4 °C for 48 h, washed four times in 1× PBS with 0.1% Triton X-100 and incubated with fluorophore-conjugated secondary antibodies (1:250) for 2 h at room temperature in the dark. After washing, samples were mounted with SlowFade Gold containing 10 µM DAPI for super-resolution microscopy.

The primary antibodies used for super resolution microscopy are as follows: anti-MLH1 raised in rabbit (laboratory code PAK176 (ref. ^108^), dilution 1:200), anti-HEI10 raised in chicken (PAK046, 1:10,000)^109^, anti-REC8 raised in rat (PAK036, 1:250)^73,110^, anti-REC8 raised in rabbit (PAK035, 1:250)^73,110^ and anti-CENH3 raised in chicken against the peptide acetyl-RTKHRVTRSQPRNQTDAC-amide (PAK008, 1:500)^73,111^. The anti-SGO2 antibody was raised in rat (PAK097, 1:200) against the peptide C-DQETESKDKPKADENEGMT and affinity purified against the peptide by the company LifeTein.

Secondary antibodies used in the study were conjugated with Abberior STAR Green, STAR Orange and STAR Red. All the secondary antibodies were used at a dilution of 1:200. Super-resolution images were acquired using an Abberior instruments platform (https://abberior-instruments.com/) with 485 nm, 561 nm and 640 nm excitation lasers for STAR Green, STAR Orange and STAR Red, respectively, and 775 nm (for STAR red and orange) STED depletion laser. Image deconvolution was performed using Huygens Essential version 25.10 (Scientific Volume Imaging, https://svi.nl/).

### Reporting summary

Further information on research design is available in the Nature Portfolio Reporting Summary linked to this article.

## Supplementary information

Supplementary Information Supplementary Figs. 1–7.

Reporting Summary

Peer Review File

Supplementary Table 1 Primers, markers and guides used in the study.

Supplementary Data 1 Golden SNPs used in this study.

Supplementary Data 2 Source data for supplementary Figs. 3–7.

## Source data

Source Data Fig. 1 Recombination frequency measured in cM using CTL3.437.55 interval or using PCR markers in single mutants compared to wild type. Source Data Fig. 3 Crossover number and positions across genome in various genotypes including NCBI-Genome assembly ASM2311539v1 (Col-CEN) coordinates that mark the position of NRZ, LRZ1 and LRZ2. Source Data Fig. 4 Crossover number and frequencies in NRZs and LRZs in wild type and mutants including high-resolution crossover position in NRZ and LRZs. Source Data Fig. 5 Recombination frequency measured in cM using CTL3.437.55 interval in double mutant populations. Source Data Fig. 6 Fertility analysis by seed counting in various genotypes. Source Data Extended Data Fig. 1 Recombination frequency measured in cM using CTL3.437.55 interval or using PCR markers in single mutants compared to wild type. The file also includes NCBI-Genome assembly ASM2311539v1 (Col-CEN) coordinates of the FTL and PCR markers. Source Data Extended Data Fig. 2 Number and position of crossovers both in wild-type Col/Ws hybrids and in the mutant *smc1 mp-172/smc1*. Source Data Extended Data Fig. 3 Crossover number in various genotypes. Source Data Extended Data Fig. 4 MLH1 and HEI10 foci counting in prophase cells in Wild type and in *spf2*. Source Data Extended Data Fig. 5 Recombination frequency in NRZ, LRZ1 and LRZ2 in wild type and mutants. Source Data Extended Data Fig. 7 Recombination frequency measured in cM using CTL3.437.55 interval in double mutant populations. Source Data Extended Data Fig. 8 Recombination frequency measured in cM using CTL3.437.55 interval in double mutant populations.

## Data Availability

The Col/L*er* Golden SNP list can be found in Supplementary Data 1. The raw sequencing data generated in this study have been deposited in the ArrayExpress EMBL-EBI database under accession codes E-MTAB-15440 (wild type, *spf2*+/− and *spf2*−/−), E-MTAB-15439 (wild type and *ctf18*−/−) and E-MTAB-15438 (*sgo2*+/− and *sgo2*−/−). Previously published datasets were deposited under accession codes E-MTAB-8165 (wild type^77^), E-MTAB-14425 (*recq4* (ref. ^80^)), E-MTAB-4657, E-MTAB-5476, E-MTAB-6577 (wild type and *cmt3* (ref. ^15^)), E-MTAB-14136 (*h2a.w.6*), E-MTAB-14137 (*h2a.w.7*) and E-MTAB-14138 (*h2a.w.6 h2a.w.7*)^45^. Source data are provided with this paper.
