# Supplementary material for: The deSUMOylase SPF2 and the cohesin regulators SGO2 and CTF18 suppress crossovers near centromeres
Source: Nat Plants. 2026 Jul 24;12(8):1498–513. doi: 10.1038/s41477-026-02329-1 (PMC13481266; doi:10.1038/s41477-026-02329-1)
Supplement: Supplementary file 1 [file 41477_2026_2329_MOESM1_ESM.pdf]

nature plants

Supplementary information https://doi.org/10.1038/s41477-026-02329-1

The deSUMOylase SPF2 and the cohesin regulators SGO2 and CTF18 suppress crossovers near centromeres

In the format provided by the authors and unedited

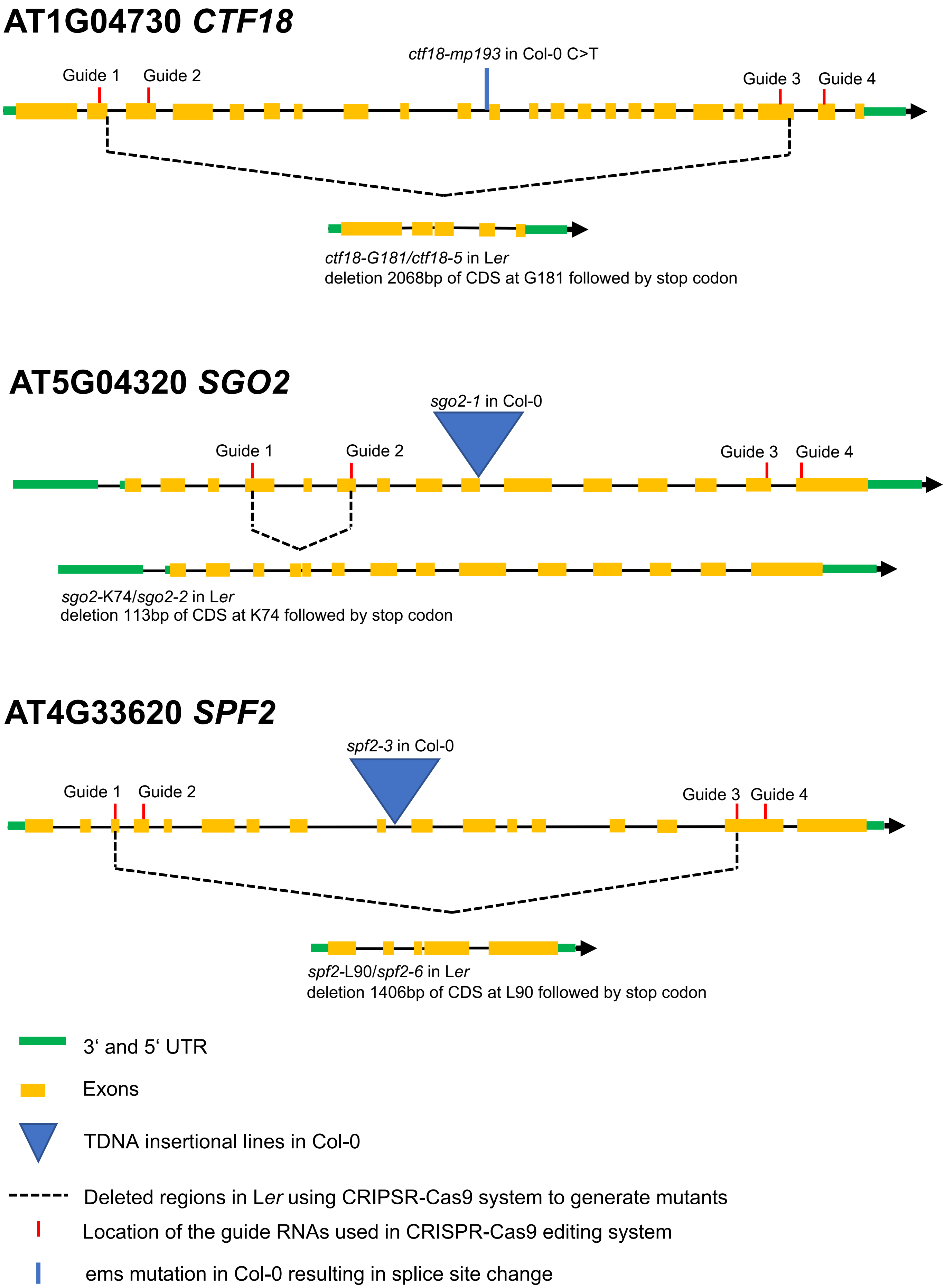

Supplementary Figure. 1. Genes and mutations used in this study

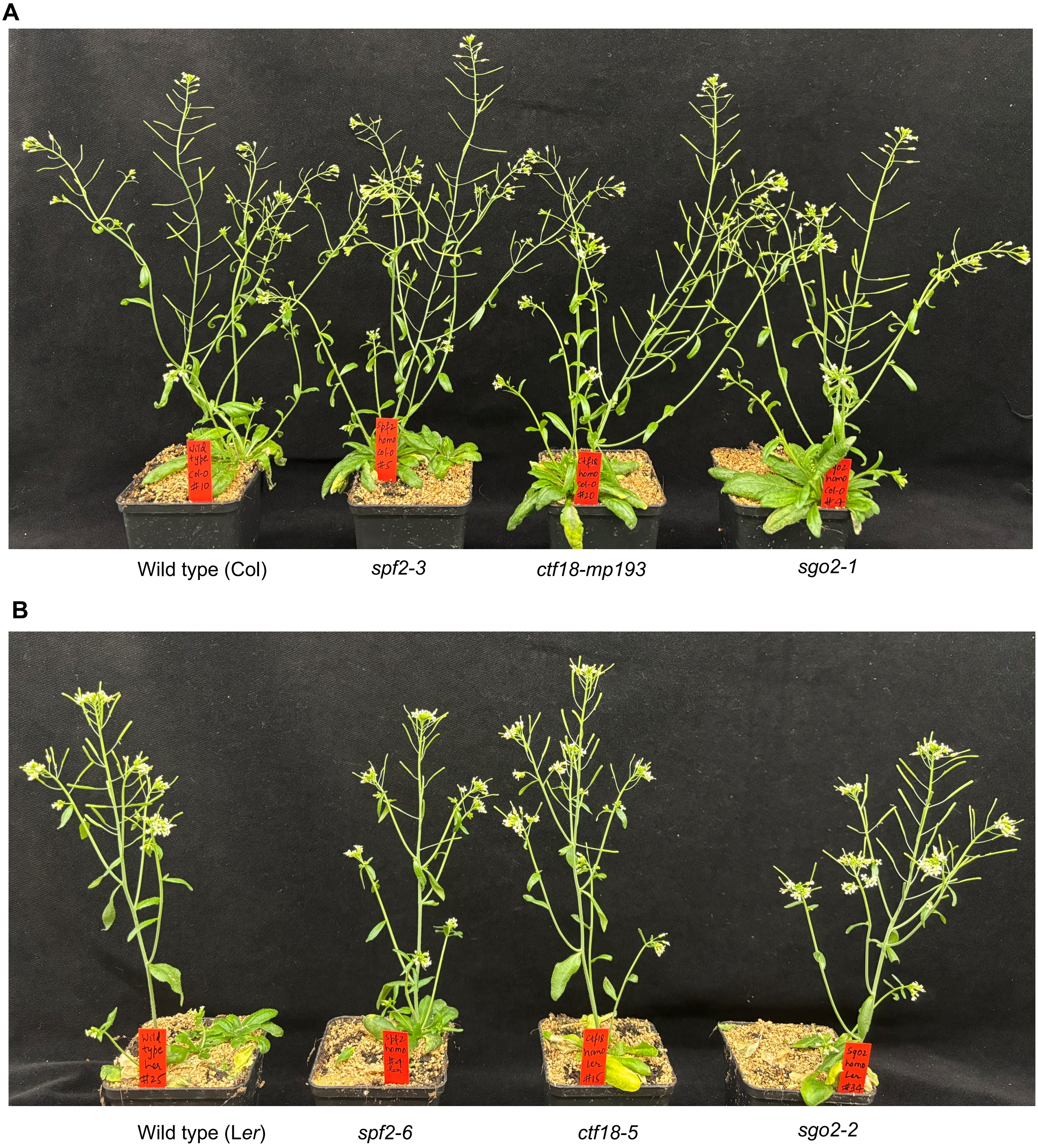

Supplementary Figure. 2. *ctf18*, *sgo2* and *spf2* mutants show growth and development indistinguishable from wild-type plants.

(A) Pictures of wild-type and mutant plants in the Col background. (B) Pictures of wild-type and mutant plants in the L*er* background

S3A

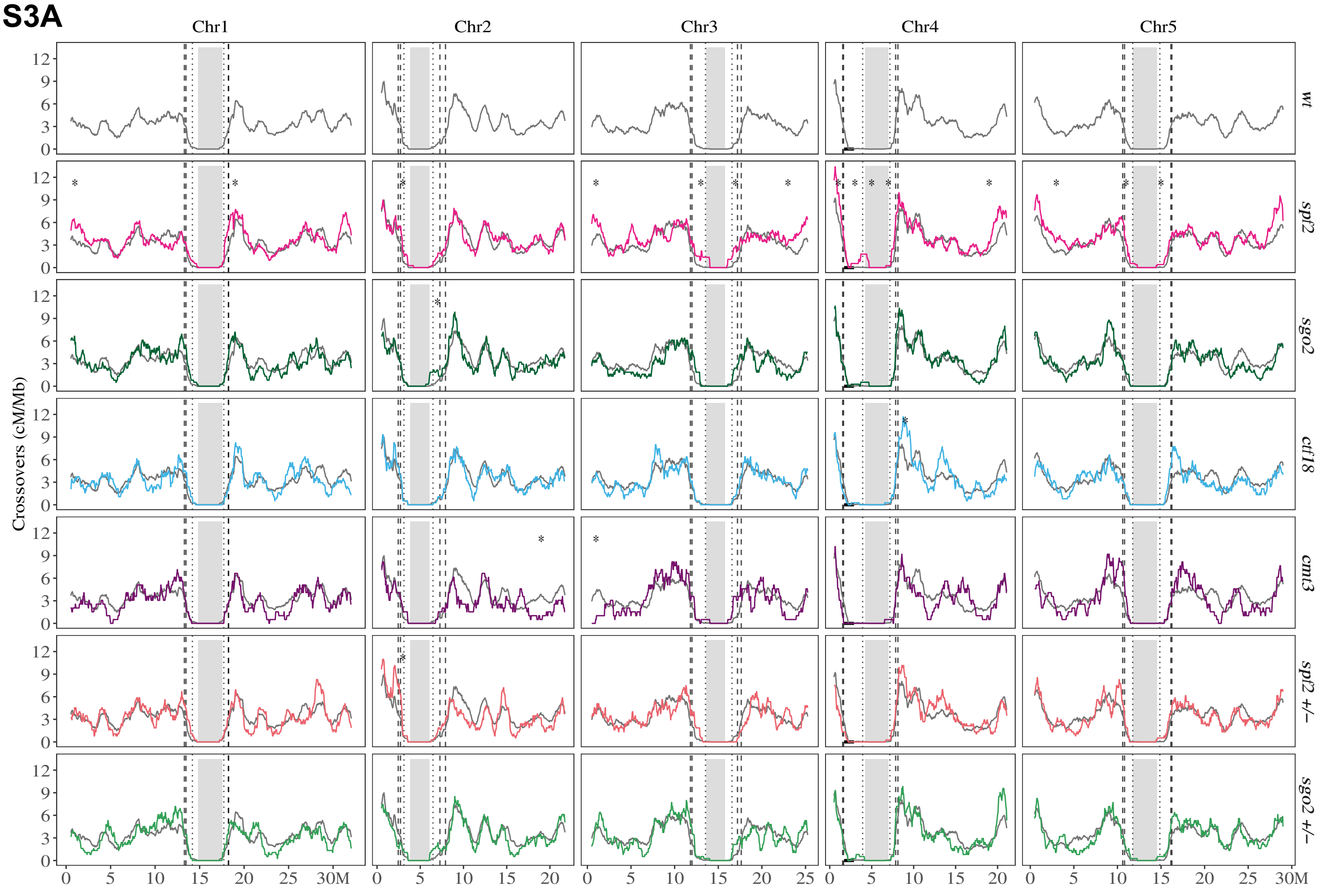

Supplementary Figure. 3. CO distribution in *spf2/+, sgo2/+, recq4a recq4b, and h2a.w.6 h2a.w.7*

(A) Genome-wide CO distribution on all 5 chromosomes in wild type (grey), *spf2 (pink), sgo2 (dark green), ctf18 (blue), cmt3 (purple)* mutants, *spf2*+/- (orange), and *sgo2*+/- (light green). Y-axis corresponds to crossovers (cM/Mb) using a 1Mb window size and 50kb step size. The X-axis corresponds to physical distance in Mb (Megabases) in the Col-CEN v1.2 assembly. The gray shading indicates the centromeric *CEN178* repeats array and the vertical dotted lines indicate the borders of the NRZ, LRZ1 and LRZ2. Asterisks indicate 2Mb intervals with a significantly different recombination rate compared to the wild type (two-sided Mann-Whitney tests, * p<0.05). The exact p values are provided in the supplementary source data.

S3B

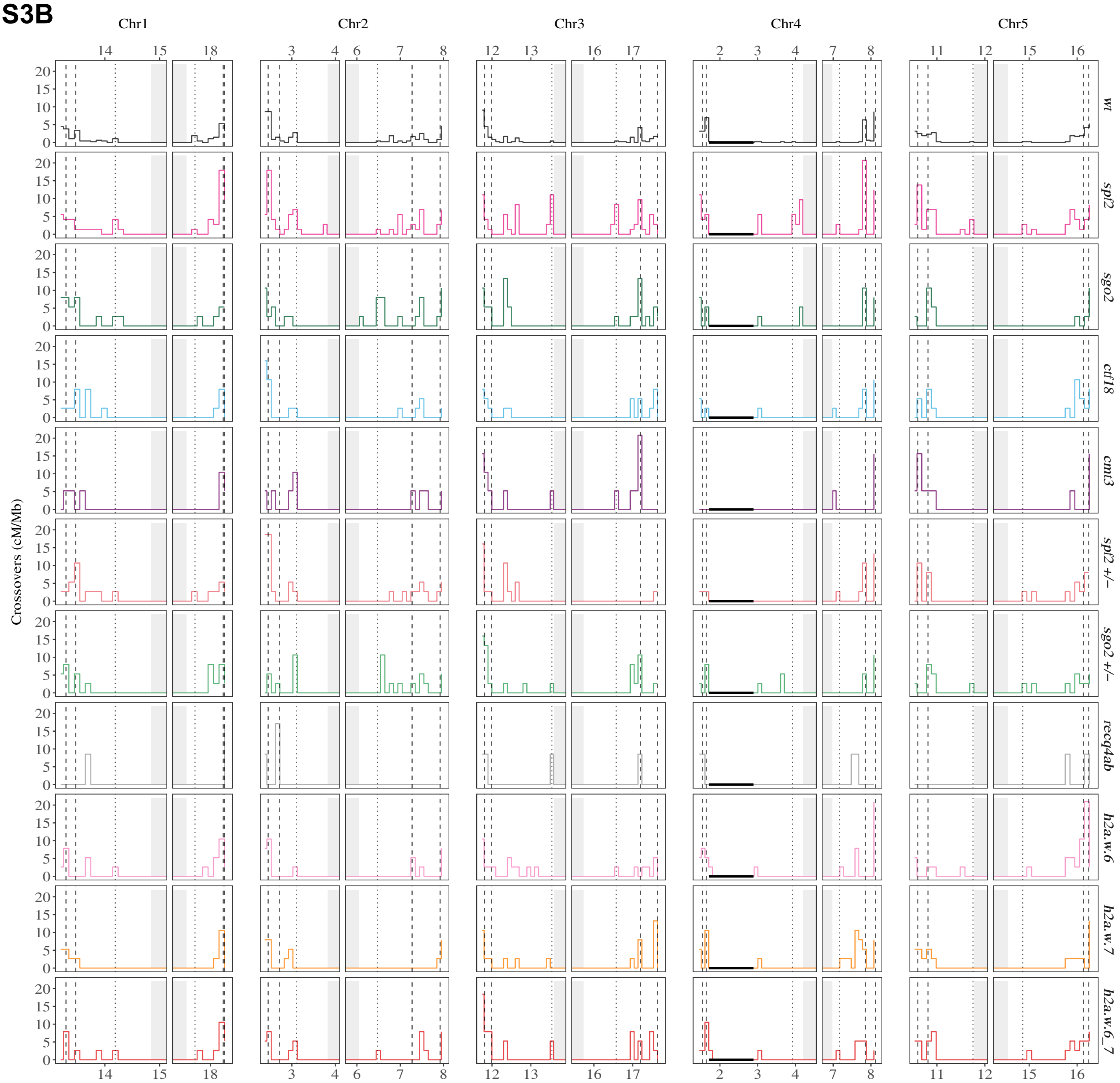

Supplementary Figure. 3. CO distribution in *spf2/+, sgo2/+, recq4a recq4b, and h2a.w.6 h2a.w.7*

(B) Crossover distribution in the centromeric region (NRZ and LRZ). Crossover frequencies are measured in non-overlapping 100kb windows. Y-axis represents crossover frequencies (cM/Mb) and X-axis corresponds to physical distance in Mb (Col-CEN). The gray shading represents the centromeric repeat array, and the vertical dotted lines the NRZ, LRZ1 and LRZ2 borders. The black horizontal line on chromosome 4 indicates the well-described inversion between Col and L*er* genomes. Supplementary source data is provided.

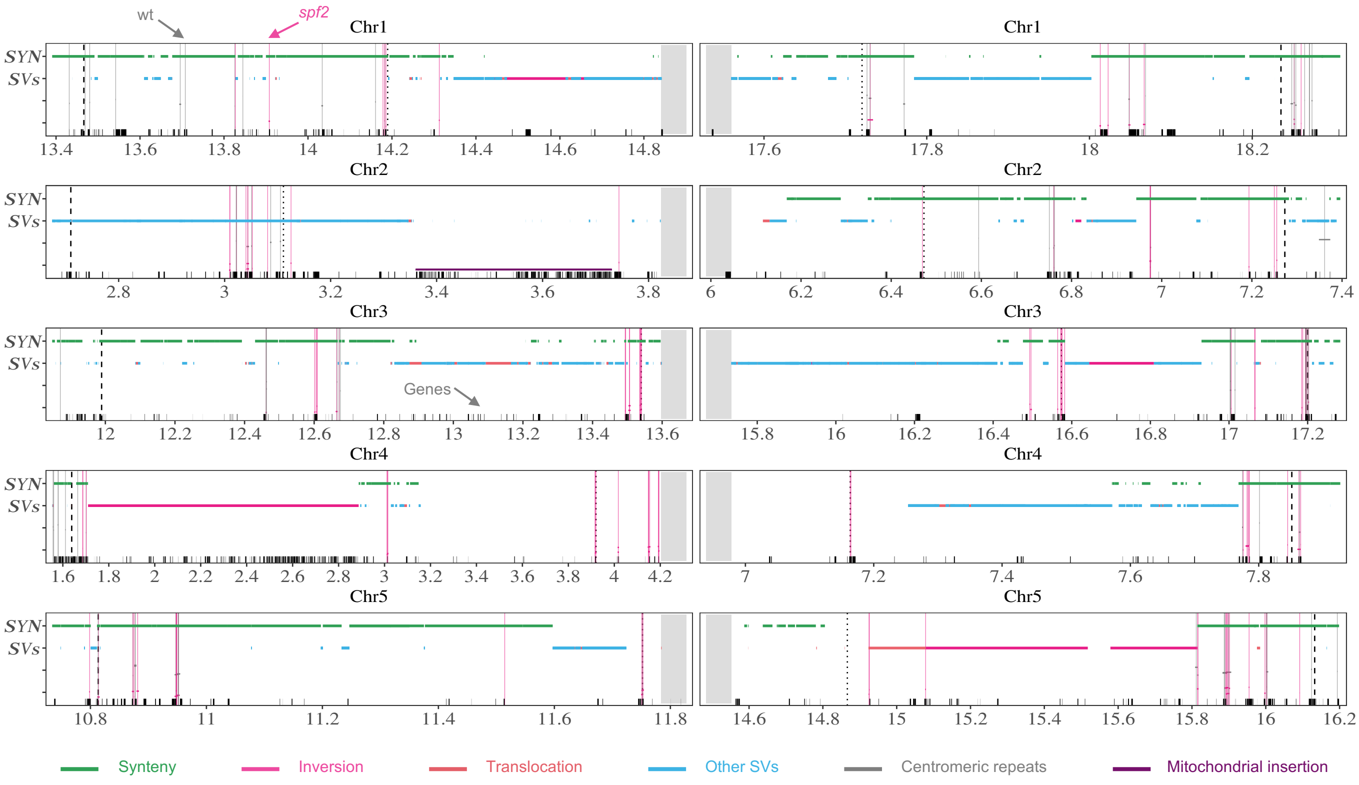

Supplementary Figure. 4. High resolution distribution of COs in NRZ and LRZ1 in wild type and *spf2*.

Related to Figure 4C. The X-axis corresponds to physical distance in Mb. Vertical lines indicate the position of COs detected in wild type (grey) and *spf2* (pink). When multiple COs occurred at overlapping positions, the counts are indicated. Grey shading shows the centromeric repeats (truncated in the middle). Horizontal bars indicate Col/L*er* syntenic regions (green), inversions (pink), translocations (red), a mitochondrial insertion (purple) and other structural variants (blue). Black ticks on the X-axis marks genes. Supplementary source data is provided.

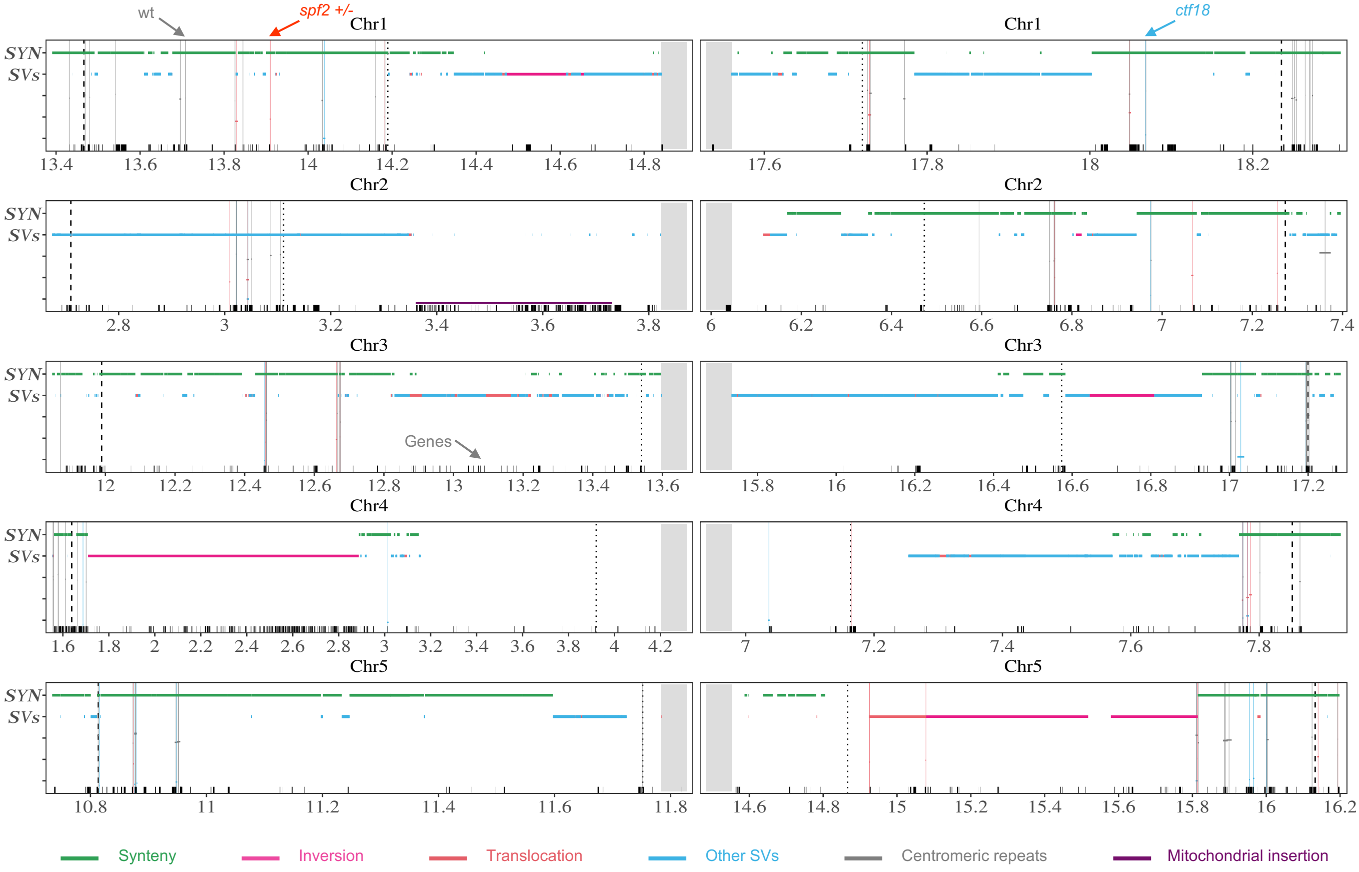

Supplementary Figure. 5. High resolution distribution of COs in NRZ and LRZ1 in *spf2+/-* and *ctf18*.

Related to Figure 4C. The X-axis corresponds to physical distance in Mb. Vertical lines indicate COs detected in wild type (grey), *spf2+/-* (orange) and *ctf18* (blue). When multiple COs occurred at overlapping positions, the counts are indicated. Grey shading shows the centromeric repeats (truncated in the middle). Horizontal bars indicate Col/L*er* syntenic regions (green), inversions (pink), translocations (red), a mitochondrial insertion (purple) and other structural variants (blue). Black ticks on the X-axis mark genes. Supplementary source data is provided.

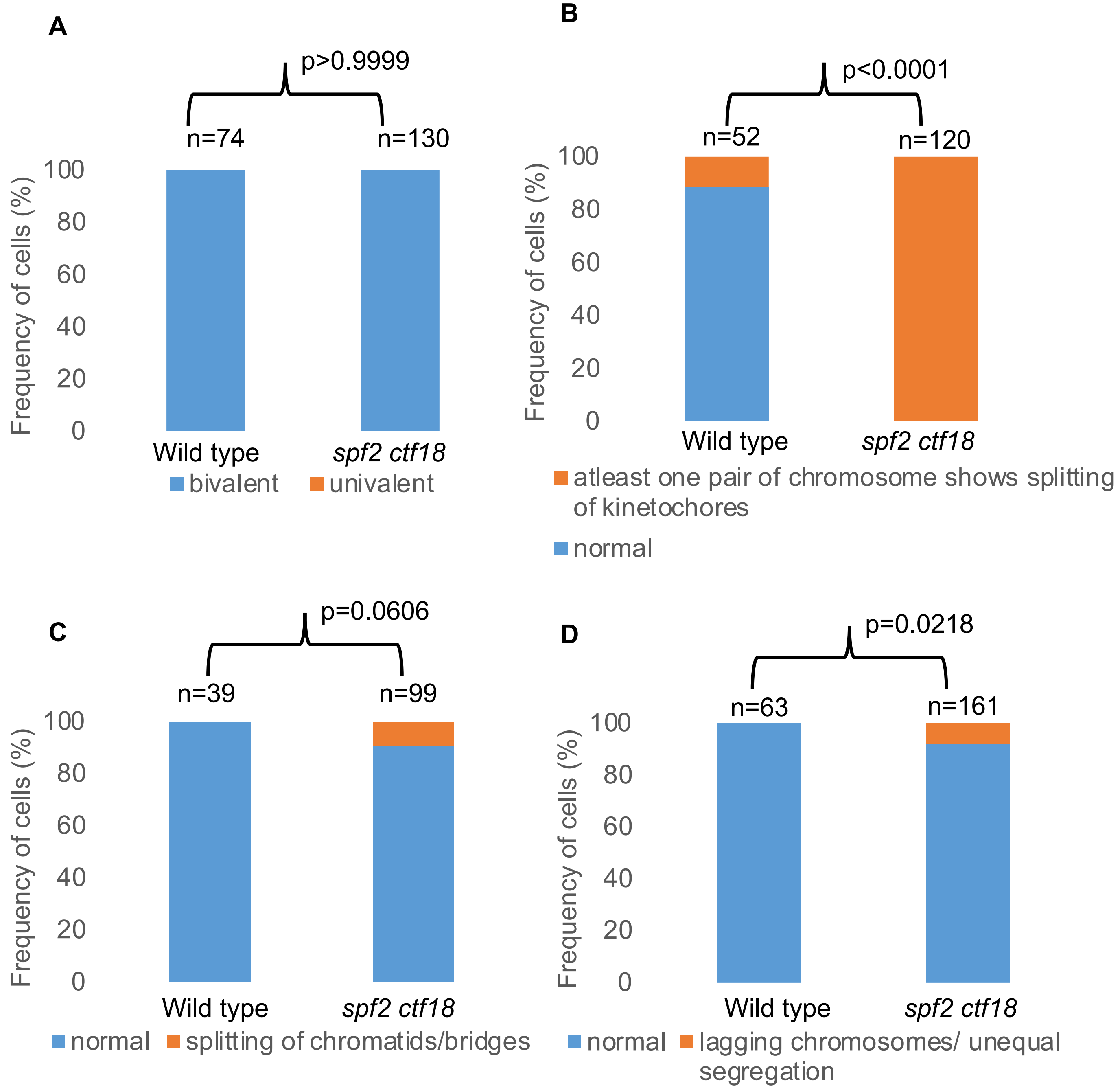

Supplementary Figure. 6. Quantification of meiotic defects in *spf2 ctf18*.

(A) Frequency of cells at metaphase I with chromosomes arranged as bivalents (physically connected by chiasmata, indicating crossover formation) or univalents (unpaired chromosomes, suggesting loss of crossover). Bivalents are shown in blue and univalents in orange. The number of cells analyzed is indicated above each bar, and statistical significance was assessed using two-sided Fisher's exact test. (B) Frequency of cells at metaphase I scored for kinetochore shape. Cells without sign of kinetochore splitting are indicated by the blue bar, while cells with at least one chromosome pair showing kinetochore splitting are indicated by the orange bar. (C) Frequency of metaphase II plates, scored for chromatid defects. Blue bar represents normal plates and orange bar indicates cells displaying premature chromatid separation or chromatid bridges. The number of analyzed metaphase II plates is indicated. (D) Frequency of anaphase II-telophase II plates, scored for segregation defects. Blue bar represents normal plates and orange bar indicates plates with unequal chromatid segregation or lagging chromosomes. The number of analyzed anaphase II plates is indicated. Mutant alleles are *ctf18-mp193* and *spf2-3.* Each experiment was performed at least three times. Supplementary source data is provided.

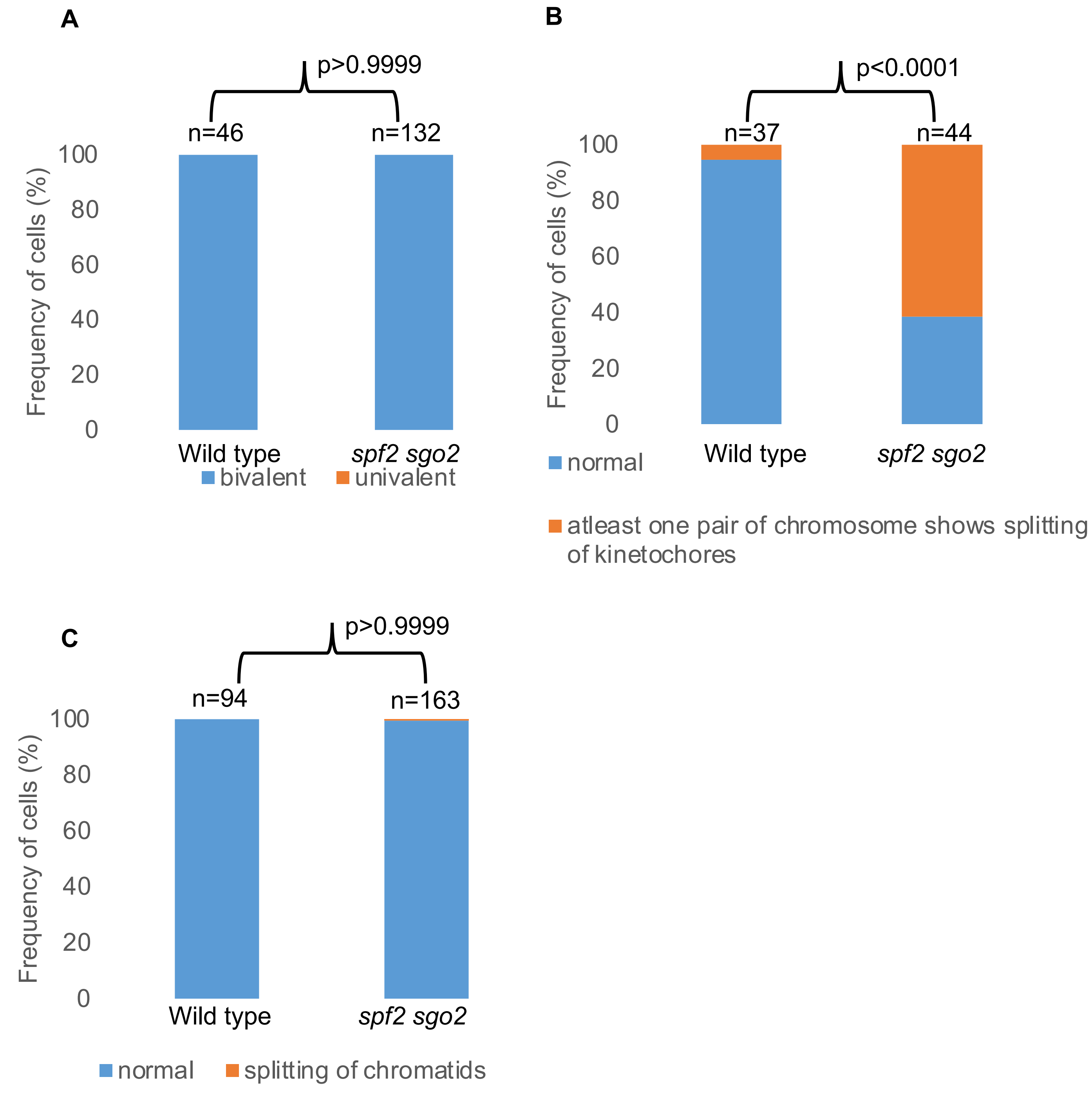

Supplementary Figure. 7. Quantification of meiotic defects in *spf2 sgo2*.

(A) Frequency of cells at metaphase I with chromosomes arranged as bivalents (physically connected by chiasmata, indicating crossover formation) or univalents (unpaired chromosomes, suggesting loss of crossover). Bivalents are shown in blue and univalents in orange. The number of cells analyzed is indicated above each bar and statistical significance was assessed using two-sided Fisher's exact test. (B) Frequency of cells at metaphase I scored for kinetochore shape. Cells without sign of kinetochore splitting are indicated by the blue bar, while cells with at least one chromosome pair showing kinetochore splitting are indicated by the orange bar. (C) Frequency of metaphase II plates, scored for chromatid defects. Blue bar represents normal plates and orange bar indicates cells displaying premature chromatid separation. The number of analyzed metaphase II plates is indicated. Mutant alleles are *sgo2-1* and *spf2-3.* Each experiment was performed at least three times. Supplementary source data is provided.
