# Supplementary material for: The deSUMOylase SPF2 and the cohesin regulators SGO2 and CTF18 suppress crossovers near centromeres
Source: Nat Plants. 2026 Jul 24;12(8):1498–513. doi: 10.1038/s41477-026-02329-1 (PMC13481266; doi:10.1038/s41477-026-02329-1)
Supplement: Supplementary file 3 [file 41477_2026_2329_MOESM3_ESM.pdf]

nature portfolio

Peer Review File

The deSUMOylase SPF2 and the cohesin regulators SGO2 and CTF18 suppress crossovers near centromeres

Corresponding Author: Professor Raphael Mercier

Version 0:

Reviewer comments:

Reviewer #1

(Comments for the Author)
Dear editor,
The authors identified that several centromeric cohesion regulators, CTF18, SGO2, and SPF2, play a role in suppressing CO formation at centromere-proximal regions in Arabidopsis thaliana. By combining analysis using FTLs and genome sequencing, they showed that functional mutations in these factors can increase CO rate at pericentromeric and/or even core-closer regions including the intervals where CO is fully off in wild-type. Further genetic studies revealed that these factors regulating CO formation near centromere parallelly to the effect of DNA methylation, opening a possibility to boost CO rate at centromeric regions via multiple genetic manipulation targets. These findings are important as they confirm the regulation of CO near centromeres by the cohesion complex, and meanwhile, they have the potential to be exploited in plant breeding programs. The manuscript is overall well-written expect for some grammar mistakes and have cited references appropriately. However, I found that the mechanistic insights are not enough based on the data sets obtained by the main approach it has applied, i.e., sequencing analysis. I have several major concerns which I would expect the authors to address in a revised manuscript before it is considered for publication in Nature Plants.
1. In addition to the reported cohesion regulators here, which other cohesion factors have been tested for CO rate increase (or decrease?) at the chromosome-wide or centromere-specific regions?
2. What’s the loading dynamic feature of CTF18, SGO2 and SPF2 during meiotic recombination in Arabidopsis? What if their localization appears much later than pachytene and/or diakinesis stages, how to explain their mutation effects on CO formation?
3. Are the increased COs at centromere-proximal regions belonging to type I or type II? Genetic and cytological experiments; e.g., combining ZMM mutants or mus81 backgrounds and immunolocalization of HEI10, are needed to clarify this question.
4. The authors did not analyze if there is any effect of an altered CO interference by an attenuated function of centromeric cohesion in those mutants. What would be the CO phenotype in those cohesion mutants in the zyp1a/b background?
5. Would an altered centromeric cohesion affect the status/ability of DSB formation, or DMC1-mediated DSB repair at the centromere-proximal regions?
6. Are the centromere-proximal regions with an increased CO number associated with alterations in DNA methylation and/or chromatin histone methylation?
The above experiments can explain the identity of the increased COs and why CO can happen in the centromere-proximal regions in the reported cohesion mutants, which will improve the importance and depth of this study.
7. The authors showed that dysfunction of those cohesion factors and an associated increased pericentromeric CO do not affect genome stability. However, this conclusion may only be appliable when plants are growing under standard conditions. Under stressful conditions, like heat, attenuated centromeric cohesion and/or occurrence of CO near centromeres may lead to defects in chromosome segregation and instability of chromosomes, which hinders the manipulating of key centromeric cohesion factors for increasing centromeric COs in crops, which grow in fields. In this case, the conclusion that the findings are promising to be applied in breeding should be tone-down.

Reviewer #2

(Comments for the Author)
Meiotic recombination is a crucial process that creates novel genetic diversity in plants. Crossovers are suppressed near centromeric regions, which make those regions inaccessible for breeding and create a major limitation for crop improvement. This work identified three genes with a suppressive effect on recombination near the centromeres: CTF18, SGO2 and SPF2. Interestingly, those genes are involved in the regulation of cohesin, and, therefore, this article points to a potential role of cohesin regulation on the suppression of recombination near the centromeres. This observation is not completely surprising given that previous work in budding and fission yeast have implicated the regulation of cohesin with crossover suppression in centromeric and pericentromeric regions. This present work suggests that a similar mechanism may exist in Arabidopsis, although the authors didn’t evaluate cohesin localisation in the mutant analysed. The strength of this article is that it identified

novel regulators of crossovers that act independently of the previously known factor, CMT3. However, some statements are not supported by the data and the article mainly describes the recombination phenotype but doesn't provide a mechanistic understanding on how recombination is suppressed by these novel factors. Overall, this article will be of interest to the field, but additional data are required to fully support the conclusions.

Specific comments:
L. 157 – 159: "This reduced recombination points to a role of cohesin in promoting CO formation". Smc1 mutant line shows lower recombination frequency in three centromeric genetic intervals. This data can be interpreted in two ways: 1) SMC1, and cohesin, promote crossover formation near the centromere, which contradicts the statement in Discussion "Our result thus suggest that proximal COs are prevented by cohesin"; or 2) total crossover level is reduced. This can be caused by a reduction in the number of DNA double strand breaks (DSBs) or the lower conversion of DSBs into COs. It seems particularly important that the authors test the two hypotheses, since one of the main focus of this article is on the potential role of cohesin on centromeric crossover formation. These hypotheses can be tested by measuring recombination in genetic intervals located on chromosome arms and at chromosome ends, and by performing a chiasma count and HEI10 foci count, in smc1 mutant.

L. 302 "Enhanced pericentromeric CO does not appear to affect genome stability" and L. 310 "Interestingly, the three trisomies were not associated with the presence of CO in the NRZ/LRZ1". This statement is not supported by the data. If a chromosome forms one crossover, one chromatid contains the recombination signature while the other chromatid lacks a signature of crossover formation. Hence, it is not possible to tell if the trisomies originated from a chromosome that had a CO near its centromere. The authors then compared the seed count per silique with the recombination rate within one centromeric interval to determine if centromeric crossovers have a general impact on fertility. This analysis using one genetic interval is not sufficient to draw such conclusions, particularly because the authors showed that not all the centromeric regions respond to the same degree to the lost of spf2, ctf18 and sgo2 in term of recombination rate. It is an important question in the field to know whether centromeric crossovers have an impact on fertility and this analysis should be performed using whole-genome crossover mapping data before any conclusions can be drawn. I recommend the authors to consider performing a sequencing of the genomes of the double mutant lines to map the crossovers genome-wide and then compare the number of crossovers in NRZ, LRZ1 and LRZ2 regions with the fertility rate.

L. 437. What is the reason for using two different methods (CellProfiler and Fiji) to measure recombination frequency in the fluorescent tagged lines? It seems that one method was used for some genetic intervals and the other method was used for the other genetic intervals.

L. 491. The authors should provide information about the method for DNA extraction, library preparation, and sequencing instruments.

Figure 2. It seems that spf2 mutation causes an increase in total crossover number. The authors should fully characterise the recombination phenotype and determine whether class I, class II, or both classes, are increased by performing a chiasma count in spf2 and spf2 x zmm mutant and immunostaining of HEI10/MLH1 in spf2.

Reviewer #3

(Comments for the Author)
A central problem of plant breeding is the segregation of linked traits during crop improvement. Genetic linkages can be broken by meiotic crossovers (COs). It is therefore of central interest to define the factors that control COs frequencies to be able to enhance genetic exchange. The group of Rafael Mercier was already able to identify in a row of seminal studies several proteins involved in DNA repair like the DNA helicase RecQ4 the mutation of which leads to a massive increase of COs in euchromatic regions. However, CO frequencies were not enhanced in heterochromatic regions including centromeres and pericentric region. These regions are almost completable devoid of COs. Nevertheless, these regions also carry genes and it would highly attractive to open these regions for genetic exchange.
In the current study the authors were able now able to identify three factors that actively limit COs around centromeres: the cohesion establishment factor CTF18, the centromeric cohesin protector SGO2, and the deSUMOylase SPF2. Mutation of these factors results in the formation of some COs in regions that were completely CO free but also modestly enhance their frequency in heterochromatic regions where they were rare before. Certain but not all combination of the mutations further increases COs frequencies. The nature of the mutated genes indicate that cohesins and the regulation of their turnovers are important for the suppressing COs in pericentromeric regions.

The is a very well done and convincing study reporting on results that are definitely of general interest, important for our basic understanding of meiosis but also for plant breeding applications.

There is only concern I have, if it comes to practical applications. Mutation of the well characterized CO suppressors RecQ4A or FancM results in somatic repair defects and hyperrecombination, which might cause unwanted secondary mutations. As far as I understand from the manuscript (I might be wrong) CTF18, SGO2, and SPF2 seem to have also important roles in somatic cells. I think the authors should elaborate on this more clearly in the discussion. The authors analyzed meiotic chromosome spreads to detect aberrations and genetic instability. It would be interesting to do the same with mitotic spreads, at least for the factors that are not meiosis-specific.

Decision Letter:

25th November 2025

Dear Professor Mercier,

Your Article, "The deSUMOylase SPF2 and the cohesin regulators SGO2 and CTF18 suppress crossovers near centromeres" has now been seen by three referees. You will see from their comments below that while they find your work of interest, some important points are raised. We are interested in the possibility of publishing your study in Nature Plants, but would like to consider your response to these concerns in the form of a revised manuscript before we make a final decision on publication.

As you can read below, all reviewers think that the findings are potentially interesting and relevant, however, more evidence is required to fully support the main conclusions. In our opinion the main points are:

1. Reviewer #1 thinks that it should be tested whether increased CO number is associated with alterations in DNA methylation and/or chromatin histone methylation, and whether increased COs at centromere-proximal regions belong to type I or type II.
2. Reviewer #2 states that the cause for reduced recombination frequency in smc1 mutants should be investigated in more detail. Furthermore, to test whether centromeric crossovers have an impact on fertility whole-genome crossover mapping data is required.
3. Reviewer #3 thinks that it should be discussed what the function of CTF18, SGO2, and SPF2 in somatic cells might be.

All referees also raise some other points that should be addressed. As usual, please do not hesitate to get in touch if you would like to discuss these issues further.

We therefore invite you to revise your manuscript taking into account all reviewer and editor comments. Please highlight all changes in the manuscript text file, preferably in Microsoft Word format.

We are committed to providing a fair and constructive peer-review process. Do not hesitate to contact us if there are specific requests from the reviewers that you believe are technically impossible or unlikely to yield a meaningful outcome.

When revising your manuscript please:

* Include a "Response to referees" document detailing, point-by-point, how you addressed each referee comment. If no action was taken to address a point, you must provide a compelling argument. This response will be sent back to the referees along with the revised manuscript.

* If you have not done so already please begin to revise your manuscript so that it conforms to our Article format instructions at http://www.nature.com/nplants/info/final-submission. Refer also to any guidelines provided in this letter.

* Include a revised version of any required reporting checklist. It will be available to referees (and, potentially, statisticians) to aid in their evaluation if the manuscript goes back for peer review. A revised checklist is essential for re-review of the paper.

* Pay close attention to our href="https://www.nature.com/nature-portfolio/editorial-policies/image-integrity">Digital Image Integrity Guidelines. and to the following points. Please ensure:
-- that unprocessed scans are clearly labelled and match the gels and western blots presented in figures.
-- that control panels for gels and western blots are appropriately described as loading on sample processing controls
-- that all images in the paper are checked for duplication of panels and for splicing of gel lanes.
-- that you retain unprocessed data and metadata files after publication, ideally archiving data in perpetuity. These may be requested during the peer review and production process or after publication if any issues arise.

EXTENDED DATA FIGURES

When re-submitting your manuscript, please ensure that any supplementary figures and tables that are crucial to the manuscript's conclusions are converted into Extended Data figures and tables to increase visibility of these data. Extended Data figures and tables are online-only (present in the online PDF and full-text HTML versions of the paper), peer-reviewed display items that provide essential background to the article but are not included in the main article due to space constraints. A maximum of ten Extended Data display items (figures and tables) is permitted.

Nature Plants strongly supports public availability of data and are therefore keen that the data used in your paper is placed in an appropriate public data repository. Alternatively, if this is not possible, you may present the data as Extended Data or Supplementary Information. If data can only be shared on request, please explain why in your Data Availability Statement, and also in the correspondence with your editor. Please note that for some data types, deposition in a public repository is mandatory.

Please use the link below to submit your revised manuscript and related files:

Link Redacted

Note: This URL links to your confidential home page and associated information about manuscripts you may have submitted, or that you are reviewing for us. If you wish to forward this email to co-authors, please delete the link to your homepage.

We hope to receive your revised manuscript within three to four months, however, this is not a strict deadline. If you cannot send it within this time, please let us know. We will be happy to consider your revision so long as nothing similar has been accepted for publication at Nature Plants or published elsewhere.

We look forward to seeing the revised manuscript and thank you for the opportunity to review your work.

Sincerely,

Nature Plants is committed to improving transparency in authorship. As part of our efforts in this direction, we are now requesting that all authors identified as 'corresponding author' on published papers create and link their Open Researcher and Contributor Identifier (ORCID) with their account on the Manuscript Tracking System (MTS), prior to acceptance. This applies to primary research papers only. ORCID helps the scientific community achieve unambiguous attribution of all scholarly contributions. You can create and link your ORCID from the home page of the MTS by clicking on 'Modify my Springer Nature account'. For more information please visit please visit www.springernature.com/orcid.

Please do not hesitate to contact me if you have any questions or would like to discuss these revisions further.

****

Reviewers' Comments:

Reviewer #1 (Comments for the Author):

The authors identified that several centromeric cohesion regulators, CTF18, SGO2, and SPF2, play a role in suppressing CO formation at centromere-proximal regions in Arabidopsis thaliana. By combining analysis using FTLs and genome sequencing, they showed that functional mutations in these factors can increase CO rate at pericentromeric and/or even core-closer regions including the intervals where CO is fully off in wild-type. Further genetic studies revealed that these factors regulating CO formation near centromere parallelly to the effect of DNA methylation, opening a possibility to boost CO rate at centromeric regions via multiple genetic manipulation targets. These findings are important as they confirm the regulation of CO near centromeres by the cohesion complex, and meanwhile, they have the potential to be exploited in plant breeding programs. The manuscript is overall well-written expect for some grammar mistakes and have cited references appropriately. However, I found that the mechanistic insights are not enough based on the data sets obtained by the main approach it has applied, i.e., sequencing analysis. I have several major concerns which I would expect the authors to address in a revised manuscript before it is considered for publication in Nature Plants.
1. In addition to the reported cohesion regulators here, which other cohesion factors have been tested for CO rate increase (or decrease?) at the chromosome-wide or centromere-specific regions?
2. What’s the loading dynamic feature of CTF18, SGO2 and SPF2 during meiotic recombination in Arabidopsis? What if their localization appears much later than pachytene and/or diakinesis stages, how to explain their mutation effects on CO formation?
3. Are the increased COs at centromere-proximal regions belonging to type I or type II? Genetic and cytological experiments; e.g., combining ZMM mutants or mus81 backgrounds and immunolocalization of HEI10, are needed to clarify this question.
4. The authors did not analyze if there is any effect of an altered CO interference by an attenuated function of centromeric cohesion in those mutants. What would be the CO phenotype in those cohesion mutants in the zyp1a/b background?
5. Would an altered centromeric cohesion affect the status/ability of DSB formation, or DMC1-mediated DSB repair at the centromere-proximal regions?
6. Are the centromere-proximal regions with an increased CO number associated with alterations in DNA methylation and/or chromatin histone methylation?
The above experiments can explain the identity of the increased COs and why CO can happen in the centromere-proximal regions in the reported cohesion mutants, which will improve the importance and depth of this study.
7. The authors showed that dysfunction of those cohesion factors and an associated increased pericentromeric CO do not affect genome stability. However, this conclusion may only be appliable when plants are growing under standard conditions. Under stressful conditions, like heat, attenuated centromeric cohesion and/or occurrence of CO near centromeres may lead to defects in chromosome segregation and instability of chromosomes, which hinders the manipulating of key centromeric cohesion factors for increasing centromeric COs in crops, which grow in fields. In this case, the conclusion that the findings are promising to be applied in breeding should be tone-down.

Reviewer #2 (Comments for the Author):

Meiotic recombination is a crucial process that creates novel genetic diversity in plants. Crossovers are suppressed near centromeric regions, which make those regions inaccessible for breeding and create a major limitation for crop improvement. This work identified three genes with a suppressive effect on recombination near the centromeres: CTF18, SGO2 and SPF2. Interestingly, those genes are involved in the regulation of cohesin, and, therefore, this article points to a potential role of cohesin regulation on the suppression of recombination near the centromeres. This observation is not completely surprising given that previous work in budding and fission yeast have implicated the regulation of cohesin with crossover suppression in

centromeric and pericentromeric regions. This present work suggests that a similar mechanism may exist in Arabidopsis, although the authors didn't evaluate cohesin localisation in the mutant analysed. The strength of this article is that it identified novel regulators of crossovers that act independently of the previously known factor, CMT3. However, some statements are not supported by the data and the article mainly describes the recombination phenotype but doesn't provide a mechanistic understanding on how recombination is suppressed by these novel factors. Overall, this article will be of interest to the field, but additional data are required to fully support the conclusions.

Specific comments:
L. 157 – 159: "This reduced recombination points to a role of cohesin in promoting CO formation". Smc1 mutant line shows lower recombination frequency in three centromeric genetic intervals. This data can be interpreted in two ways: 1) SMC1, and cohesin, promote crossover formation near the centromere, which contradicts the statement in Discussion "Our result thus suggest that proximal COs are prevented by cohesin"; or 2) total crossover level is reduced. This can be caused by a reduction in the number of DNA double strand breaks (DSBs) or the lower conversion of DSBs into COs. It seems particularly important that the authors test the two hypotheses, since one of the main focus of this article is on the potential role of cohesin on centromeric crossover formation. These hypotheses can be tested by measuring recombination in genetic intervals located on chromosome arms and at chromosome ends, and by performing a chiasma count and HEI10 foci count, in smc1 mutant.

L. 302 "Enhanced pericentromeric CO does not appear to affect genome stability" and L. 310 "Interestingly, the three trisomies were not associated with the presence of CO in the NRZ/LRZ1". This statement is not supported by the data. If a chromosome forms one crossover, one chromatid contains the recombination signature while the other chromatid lacks a signature of crossover formation. Hence, it is not possible to tell if the trisomies originated from a chromosome that had a CO near its centromere. The authors then compared the seed count per silique with the recombination rate within one centromeric interval to determine if centromeric crossovers have a general impact on fertility. This analysis using one genetic interval is not sufficient to draw such conclusions, particularly because the authors showed that not all the centromeric regions respond to the same degree to the lost of spf2, ctf18 and sgo2 in term of recombination rate. It is an important question in the field to know whether centromeric crossovers have an impact on fertility and this analysis should be performed using whole-genome crossover mapping data before any conclusions can be drawn. I recommend the authors to consider performing a sequencing of the genomes of the double mutant lines to map the crossovers genome-wide and then compare the number of crossovers in NRZ, LRZ1 and LRZ2 regions with the fertility rate.

L. 437. What is the reason for using two different methods (CellProfiler and Fiji) to measure recombination frequency in the fluorescent tagged lines? It seems that one method was used for some genetic intervals and the other method was used for the other genetic intervals.

L. 491. The authors should provide information about the method for DNA extraction, library preparation, and sequencing instruments.

Figure 2. It seems that spf2 mutation causes an increase in total crossover number. The authors should fully characterise the recombination phenotype and determine whether class I, class II, or both classes, are increased by performing a chiasma count in spf2 and spf2 x zmm mutant and immunostaining of HEI10/MLH1 in spf2.

Reviewer #3 (Comments for the Author):

A central problem of plant breeding is the segregation of linked traits during crop improvement. Genetic linkages can be broken by meiotic crossovers (COs). It is therefore of central interest to define the factors that control COs frequencies to be able to enhance genetic exchange. The group of Rafael Mercier was already able to identify in a row of seminal studies several proteins involved in DNA repair like the DNA helicase RecQ4 the mutation of which leads to a massive increase of COs in euchromatic regions. However, CO frequencies were not enhanced in heterochromatic regions including centromeres and pericentric region. These regions are almost completable devoid of COs. Nevertheless, these regions also carry genes and it would highly attractive to open these regions for genetic exchange.
In the current study the authors were able now able to identify three factors that actively limit COs around centromeres: the cohesion establishment factor CTF18, the centromeric cohesin protector SGO2, and the deSUMOylase SPF2. Mutation of these factors results in the formation of some COs in regions that were completely CO free but also modestly enhance their frequency in heterochromatic regions where they were rare before. Certain but not all combination of the mutations further increases COs frequencies. The nature of the mutated genes indicate that cohesins and the regulation of their turnovers are important for the suppressing COs in pericentromeric regions.

The is a very well done and convincing study reporting on results that are definitely of general interest, important for our basic understanding of meiosis but also for plant breeding applications.

There is only concern I have, if it comes to practical applications. Mutation of the well characterized CO suppressors RecQ4A or FancM results in somatic repair defects and hyperrecombination, which might cause unwanted secondary mutations. As far as I understand from the manuscript (I might be wrong) CTF18, SGO2, and SPF2 seem to have also important roles in somatic cells. I think the authors should elaborate on this more clearly in the discussion. The authors analyzed meiotic chromosome spreads to detect aberrations and genetic instability. It would be interesting to do the same with mitotic spreads, at least for the factors that are not meiosis-specific.

********************END********************

Version 1:

Reviewer comments:

Reviewer #1

(Comments for the Author)
Dear editor,
The authors have tried to address my concerns. Although in the revised manuscript they did not address whether DSB formation and chromatin epigenetic mdofications at the centromere-proximal regions are altered in the analyzed mutants, which were requested in my first-round review, they have discussed the potential mechanisms properly. In the revised manuscript, they analyzed the chromosome localization of SGO2 and provided further insights into the CO distribution in the mutants by sequencing approach. Actually, I still would like very much to know more about how these cohesion factors mechanistically influence CO near the centromeres, while, I understand that those need much more time and work and thus will delay the report of the function of SPF2, SGO2 and CTF18. Finally, I am satisfied with the revised version of the manuscript.

Reviewer #2

(Comments for the Author)
The authors included new data in the manuscript. These new data strengthen the study and will be of interest to the meiosis community. The authors addressed my comments and I don't have further comments.

Reviewer #3

(Comments for the Author)
The authors took up my concern and discussed it a proper way at the end of the article. For me the revised version is of general interest to the plant community.

Decision Letter:

Our ref: NPLANTS-251021170A

30th April 2026

Dear Dr. Mercier,

Thank you for submitting your revised manuscript "The deSUMOylase SPF2 and the cohesin regulators SGO2 and CTF18 suppress crossovers near centromeres" (NPLANTS-251021170A). It has now been seen by the original referees and their comments are below. The reviewers find that the paper has improved in revision, and therefore we'll be happy in principle to publish it in Nature Plants, pending minor revisions to satisfy the referees' final requests and to comply with our editorial and formatting guidelines.

We are now performing detailed checks on your paper and will send you a checklist detailing our editorial and formatting requirements in about a week. Please do not upload the final materials and make any revisions until you receive this additional information from us.

Thank you again for your interest in Nature Plants Please do not hesitate to contact me if you have any questions.

Reviewer #1 (Comments for the Author):

Dear editor,
The authors have tried to address my concerns. Although in the revised manuscript they did not address whether DSB formation and chromatin epigenetic mdofications at the centromere-proximal regions are altered in the analyzed mutants, which were requested in my first-round review, they have discussed the potential mechanisms properly. In the revised manuscript, they analyzed the chromosome localization of SGO2 and provided further insights into the CO distribution in the mutants by sequencing approach. Actually, I still would like very much to know more about how these cohesion factors mechanistically influence CO near the centromeres, while, I understand that those need much more time and work and thus will delay the report of the function of SPF2, SGO2 and CTF18. Finally, I am satisfied with the revised version of the

manuscript.

Reviewer #2 (Comments for the Author):

The authors included new data in the manuscript. These new data strengthen the study and will be of interest to the meiosis community. The authors addressed my comments and I don't have further comments.

Reviewer #3 (Comments for the Author):

The authors took up my concern and discussed it a proper way at the end of the article. For me the revised version is of general interest to the plant community.

Version 2:

Decision Letter:

2nd June 2026

Dear Professor Mercier,

We are pleased to inform you that your Article entitled "The deSUMOylase SPF2 and the cohesin regulators SGO2 and CTF18 suppress crossovers near centromeres", has now been accepted for publication in Nature Plants.

Over the next few weeks, your paper will be copyedited to ensure that it conforms to Nature Plants style. We look particularly carefully at the titles of all papers to ensure that they are relatively brief and understandable.

Once your paper is typeset, you will receive an email with a link to choose the appropriate publishing options for your paper and our Author Services team will be in touch regarding any additional information that may be required.

After the grant of rights is completed, you will receive a link to your electronic proof via email with a request to make any corrections within 48 hours. If, when you receive your proof, you cannot meet this deadline, please inform us at rjsproduction@springernature.com immediately.

You will not receive your proofs until the publishing agreement has been received through our system.

You may wish to make your media relations office aware of your accepted publication, in case they consider it appropriate to organize some internal or external publicity. Once your paper has been scheduled you will receive an email confirming the publication details. This is normally 3-4 working days in advance of publication. If you need additional notice of the date and time of publication, please let the production team know when you receive the proof of your article to ensure there is sufficient time to coordinate. Further information on our embargo policies can be found here:
https://www.nature.com/authors/policies/embargo.html

Due to the importance of these deadlines, we ask you please us know now whether you will be difficult to contact over the next month. If this is the case, we ask you provide us with the contact information (email, phone and fax) of someone who will be able to check the proofs on your behalf, and who will be available to address any last-minute problems.

Acceptance of your manuscript is conditional on all authors' agreement with our publication policies (see http://www.nature.com/authors/policies/index.html). In particular your manuscript must not be published elsewhere.

If you have any questions about our publishing options, costs, Open Access requirements, or our legal forms, please contact ASJournals@springernature.com

Authors may need to take specific actions to achieve compliance with funder and institutional open access mandates. If your research is supported by a funder that requires immediate open access (e.g. according to Plan S principles or the NIH public access policy) then you should select the gold OA route, and we will direct you to the compliant route where possible. Because authors warrant under our subscription licensing terms that they haven't committed to licensing any version of their article under a licence inconsistent with the terms of our agreement – including the applicable embargo period – publication under the subscription model isn't suitable for authors whose funders require no embargo.

An online order form for reprints of your paper is available at https://www.nature.com/reprints/author-reprints.html. All co-authors, authors' institutions and authors'

funding agencies can order reprints using the form appropriate to their geographical region.

We welcome the submission of potential cover material (including a short caption of around 40 words) related to your manuscript; suggestions should be sent to Nature Plants as electronic files (the image should be 300 dpi at 210 x 297 mm in either TIFF or JPEG format). Please note that such pictures should be selected more for their aesthetic appeal than for their scientific content, and that colour images work better than black and white or grayscale images. Please include a written description of your image and how it was created along with your image files. Please do not try to design a cover with the Nature Plants logo etc., and please do not submit composites of images related to your work. I am sure you will understand that we cannot make any promise as to whether any of your suggestions might be selected for the cover of the journal.

You can now use a single sign-on for all your accounts, view the status of all your manuscript submissions and reviews, access usage statistics for your published articles and download a record of your refereeing activity for the Nature journals.

To assist our authors in disseminating their research to the broader community, our SharedIt initiative provides you with a unique shareable link that will allow anyone (with or without a subscription) to read the published article. Recipients of the link with a subscription will also be able to download and print the PDF.

As soon as your article is published, you will receive an automated email with your shareable link.

P.S. Click on the following link if you would like to recommend Nature Plants to your librarian
http://www.nature.com/subscriptions/recommend.html#forms

** Visit the Springer Nature Editorial and Publishing website at www.springernature.com/editorial-and-publishing-jobs for more information about our career opportunities. If you have any questions please click here.**

Open Access This Peer Review File is licensed under a Creative Commons Attribution 4.0 International License, which permits use, sharing, adaptation, distribution and reproduction in any medium or format, as long as you give appropriate credit to the original author(s) and the source, provide a link to the Creative Commons license, and indicate if changes were made. In cases where reviewers are anonymous, credit should be given to 'Anonymous Referee' and the source.
The images or other third party material in this Peer Review File are included in the article's Creative Commons license, unless indicated otherwise in a credit line to the material. If material is not included in the article's Creative Commons license and your intended use is not permitted by statutory regulation or exceeds the permitted use, you will need to obtain permission directly from the copyright holder.
To view a copy of this license, visit https://creativecommons.org/licenses/by/4.0/

Reviewer #1 (Comments for the Author):

The authors identified that several centromeric cohesion regulators, CTF18, SGO2, and SPF2, play a role in suppressing CO formation at centromere-proximal regions in Arabidopsis thaliana. By combining analysis using FTLs and genome sequencing, they showed that functional mutations in these factors can increase CO rate at pericentromeric and/or even core-closer regions including the intervals where CO is fully off in wild-type. Further genetic studies revealed that these factors regulating CO formation near centromere parallelly to the effect of DNA methylation, opening a possibility to boost CO rate at centromeric regions via multiple genetic manipulation targets. These findings are important as they confirm the regulation of CO near centromeres by the cohesion complex, and meanwhile, they have the potential to be exploited in plant breeding programs. The manuscript is overall well-written expect for some grammar mistakes and have cited references appropriately. However, I found that the mechanistic insights are not enough based on the data sets obtained by the main approach it has applied, i.e., sequencing analysis. I have several major concerns which I would expect the authors to address in a revised manuscript before it is considered for publication in Nature Plants.

> Thank you for evaluating the manuscript. While we agree that our current understanding of the mechanism is incomplete, we believe that the discovery of factors preventing crossover next to the centromere is significant enough to be shared at this time. This would also enable other teams to contribute to a better understanding of the mechanisms and to test the mutations in crop species. We have also included a number of new functional data in the revised manuscript, as described below.

1. In addition to the reported cohesion regulators here, which other cohesion factors have been tested for CO rate increase (or decrease?) at the chromosome-wide or centromere-specific regions?

> We did not test other regulators that the one shown in Figure 1 (*cenpc, smc3, ctf18, sgo2, spf2 and smc1*). We clarified this point in the revised manuscript. Please note that cohesin and kinetochore mutants are typically lethal and we benefited here from specific alleles (*cenc-mp221, smc3-mp226, smc1-mp172*) identified in a screen for monopolar orientation, which are not loss of function and thus are viable.

2. What's the loading dynamic feature of CTF18, SGO2 and SPF2 during meiotic recombination in Arabidopsis? What if their localization appears much later than pachytene and/or diakinesis stages, how to explain their mutation effects on CO formation?

> CTF18 is a conserved regulator of cohesion established at replication, thus prior to recombination. It is reasonable to propose that its absence modify chromosome organization and thus the recombination process. We included this consideration in the revised manuscript. The loading dynamic of SPF2 is unknown, and we unfortunately failed to produce an antibody against this protein. However, we successfully raised an antibody against SGO2. Immunolocalization revealed a peri-centromeric signal at meiosis, which is absent in the *sgo2* mutant, establishing its specificity. SGO2 is present throughout prophase, including zygotene and pachytene, which is compatible with the proposed role in regulation crossover formation at pericentromeres. We have included these results in the revised manuscript (Figure 2).

3. Are the increased COs at centromere-proximal regions belonging to type I or type II? Genetic and cytological experiments; e.g., combining ZMM mutants or mus81 backgrounds and immunolocalization of HEI10, are needed to clarify this question.

> We agree that this is an interesting question. In *ctf18* and *sgo2*, the global number of crossovers is not increased, making the suggested experiments very challenging. However, in *spf2*, which has the strongest effect on peri-centromeric recombination, we also observed a global increase of CO frequency

(Figure3 A, +16%, $p<10^{-6}$). We then counted the number of HEI10/MLH1 co-foci in *spf2* and observed an increase of the mean foci number of 16% (figure S6). This shows that the CO which are increased in *spf2* are class I COs. This result and its discussion are included in the revised manuscript.

4. The authors did not analyze if there is any effect of an altered CO interference by an attenuated function of centromeric cohesion in those mutants. What would be the CO phenotype in those cohesion mutants in the zyp1a/b background?

> These are interesting question that we aim to address in the future. Combining mutation in a hybrid background and analyzing the CO distribution in the progeny of the multi-mutant hybrids requires several plant generations and is thus beyond the scope of the present work.

5. Would an altered centromeric cohesion affect the status/ability of DSB formation, or DMC1-mediated DSB repair at the centromere-proximal regions?

> This is an interesting question, but we are missing technical means to address it.

6. Are the centromere-proximal regions with an increased CO number associated with alterations in DNA methylation and/or chromatin histone methylation?

The above experiments can explain the identity of the increased COs and why CO can happen in the centromere-proximal regions in the reported cohesion mutants, which will improve the importance and depth of this study.

> Quantifying DNA methylation and/or chromatin histone modifications at meiosis is technically challenging. We are working on it but did not manage so far to have conclusive results.

7. The authors showed that dysfunction of those cohesion factors and an associated increased pericentromeric CO do not affect genome stability. However, this conclusion may only be appliable when plants are growing under standard conditions. Under stressful conditions, like heat, attenuated centromeric cohesion and/or occurrence of CO near centromeres may lead to defects in chromosome segregation and instability of chromosomes, which hinders the manipulating of key centromeric cohesion factors for increasing centromeric COs in crops, which grow in fields. In this case, the conclusion that the findings are promising to be applied in breeding should be tone-down.

> This is indeed possible that some of these mutants could express additional phenotypes under harsh conditions. However, we would like to mention that the suggested usage in breeding of these mutations does not imply that the mutations will be present in the field. Indeed, increasing crossover in pericentromeric regions would be useful to introgress traits from non-elite source (e.g. related wild-species) into elite varieties. This occurs in the early phase of a breeding scheme, and the mutation(s), after having allowed the desired recombination events, will be likely segregated out together with undesired genomic regions from the source, and thus not present in the final varieties and thus not in the fields. In this context, a reasonable penalty on growth or fertility associated with the mutation is not problematic for its usage in breeding. This has been confirmed in discussions between the senior author of this study and several plant breeders. We added these considerations in the discussion of the revised manuscript.

Reviewer #2 (Comments for the Author):

Meiotic recombination is a crucial process that creates novel genetic diversity in plants. Crossovers are suppressed near centromeric regions, which make those regions inaccessible for breeding and create a major limitation for crop improvement. This work identified three genes with a suppressive effect on recombination near the centromeres: CTF18, SGO2 and SPF2. Interestingly, those genes are involved in the regulation of cohesin, and, therefore, this article points to a potential role of cohesin regulation on the suppression of recombination near the centromeres. This observation is not completely surprising given that previous work in budding and fission yeast have implicated the regulation of cohesin with crossover suppression in centromeric and pericentromeric regions. This present work suggests that a similar mechanism may exist in Arabidopsis, although the authors didn't evaluate cohesin localisation in the mutant analysed. The strength of this article is that it identified novel regulators of crossovers that act independently of the previously known factor, CMT3. However, some statements are not supported by the data and the article mainly describes the recombination phenotype but doesn't provide a mechanistic understanding on how recombination is suppressed by these novel factors. Overall, this article will be of interest to the field, but additional data are required to fully support the conclusions.

> Thank you for your positive evaluation of the work. While we agree that our current understanding of the mechanism is incomplete, we believe that the discovery of factors preventing crossover next to the centromere is significant enough to be shared at this time. This would also enable other teams to contribute to a better understanding of the mechanisms and to test the mutations in crop species. We have also included some new functional data as described above and below.

Specific comments:

L. 157 – 159: "This reduced recombination points to a role of cohesin in promoting CO formation". Smc1 mutant line shows lower recombination frequency in three centromeric genetic intervals. This data can be interpreted in two ways: 1) SMC1, and cohesin, promote crossover formation near the centromere, which contradicts the statement in Discussion "Our result thus suggest that proximal COs are prevented by cohesin"; or 2) total crossover level is reduced. This can be caused by a reduction in the number of DNA double strand breaks (DSBs) or the lower conversion of DSBs into COs. It seems particularly important that the authors test the two hypotheses, since one of the main focus of this article is on the potential role of cohesin on centromeric crossover formation. These hypotheses can be tested by measuring recombination in genetic intervals located on chromosome arms and at chromosome ends, and by performing a chiasma count and HEI10 foci count, in smc1 mutant.

> Indeed, cohesins have also a supportive role in CO formation. Cohesins, notably REC8 and SCC3, are essential for meiotic DSB repair, and promote CO formation in Arabidopsis. In the other hand, our data (and previous work in other organisms) suggest an anti-CO role which is revealed in mild mutants. To clarify the effect of the *smc1-mp172* mutation, we performed genome-wide analysis of CO formation through progeny sequencing. The data (Figure S4) show that:

- The total CO number is not reduced in *smc1-mp172* but slightly increased compare to wild-type (9 vs 8.5 COs/F2, p=0.02).

- The distribution of CO is shifted to distal regions, with reduced CO frequency in the large peri-centromere (confirming the initial observation) and increased COs in more distal regions.

This adds to the complex relationship between cohesins and CO formation. This likely reflects the multiple roles of cohesins in genome organization, supporting both cohesion of replicated chromatid and chromatin folding through loop formation. We discuss these points in the revised manuscript.

L. 302 "Enhanced pericentromeric CO does not appear to affect genome stability" and L. 310 "Interestingly, the three trisomies were not associated with the presence of CO in the NRZ/LRZ1". This statement is not supported by the data. If a chromosome forms one crossover, one chromatid contains

the recombination signature while the other chromatid lacks a signature of crossover formation. Hence, it is not possible to tell if the trisomies originated from a chromosome that had a CO near its centromere.

> We agree. Thank you for pointing this out. A CO affects two of the four chromatids of a bivalent. A trisomy inherits two of the chromatids. If the trisomic plant inherits 1 recombinant chromatid and 1 non-recombinant, the CO would be detected. If the trisomic plant inherits two non-recombinant chromatids, the CO would be obviously not visible. If the trisomic plant inherits the two recombinant chromatids, the CO would be also invisible as they are reciprocal events. We have edited the manuscript to include these considerations.

The authors then compared the seed count per silique with the recombination rate within one centromeric interval to determine if centromeric crossovers have a general impact on fertility. This analysis using one genetic interval is not sufficient to draw such conclusions, particularly because the authors showed that not all the centromeric regions respond to the same degree to the lost of spf2, ctf18 and sgo2 in term of recombination rate. It is an important question in the field to know whether centromeric crossovers have an impact on fertility and this analysis should be performed using whole-genome crossover mapping data before any conclusions can be drawn. I recommend the authors to consider performing a sequencing of the genomes of the double mutant lines to map the crossovers genome-wide and then compare the number of crossovers in NRZ, LRZ1 and LRZ2 regions with the fertility rate.

> Genome wide mapping in hybrids multiple mutants would be of great interest but would require multiple plant generation. This is planned for the future, but is beyond the scope of the current manuscript. We have tone down our conclusion in the revised manuscript concerning the effect of proximal CO on genome stability.

L. 437. What is the reason for using two different methods (CellProfiler and Fiji) to measure recombination frequency in the fluorescent tagged lines? It seems that one method was used for some genetic intervals and the other method was used for the other genetic intervals.

> We initially applied a method established in our group, using FiJi. In this method, seeds are plated on agarose, which make them to separate from each other during imbibition. This provides high quality and easy to analyze images. However, the throughput was limited and we analyzed on average only 562 seeds per plant. We then decided to switch to the previously established protocol that combine CellProfiler and direct analysis of dry seeds. This enabled the analysis of a substantially larger number of seeds, with an average of 2,455 seeds per plant in our datasets. Importantly, recombination frequency estimates obtained using both approaches were consistent. Therefore, given its higher throughput and the larger number of seeds analyzed per plant, we used the CellProfiler method for all subsequent downstream analyses.

L. 491. The authors should provide information about the method for DNA extraction, library preparation, and sequencing instruments.

> We provide this information in the revised manuscript.

Figure 2. It seems that spf2 mutation causes an increase in total crossover number. The authors should fully characterise the recombination phenotype and determine whether class I, class II, or both classes, are increased by performing a chiasma count in spf2 and spf2 x zmm mutant and immunostaining of HEI10/MLH1 in spf2.

> Indeed, in *spf2*, we observed a global increase of CO frequency (figure 2A/revised figure3 A, +16%, $p<10^{-6}$). We then counted the number of HEI10/MLH1 co-foci in *spf2* and observed an increase of the mean foci number of 16% (Figure S4). This shows that SPF2 limits class I crossover. This result and its discussion are included in the revised manuscript.

Reviewer #3 (Comments for the Author):

A central problem of plant breeding is the segregation of linked traits during crop improvement. Genetic linkages can be broken by meiotic crossovers (COs). It is therefore of central interest to define the factors that control COs frequencies to be able to enhance genetic exchange. The group of Rafael Mercier was already able to identify in a row of seminal studies several proteins involved in DNA repair like the DNA helicase RecQ4 the mutation of which leads to a massive increase of COs in euchromatic regions. However, CO frequencies were not enhanced in heterochromatic regions including centromeres and pericentric region. These regions are almost completable devoid of COs. Nevertheless, these regions also carry genes and it would highly attractive to open these regions for genetic exchange. In the current study the authors were able now able to identify three factors that actively limit COs around centromeres: the cohesion establishment factor CTF18, the centromeric cohesin protector SGO2, and the deSUMOylase SPF2. Mutation of these factors results in the formation of some COs in regions that were completely CO free but also modestly enhance their frequency in heterochromatic regions where they were rare before. Certain but not all combination of the mutations further increases COs frequencies. The nature of the mutated genes indicate that cohesins and the regulation of their turnovers are important for the suppressing COs in pericentromeric regions.

The is a very well done and convincing study reporting on results that are definitely of general interest, important for our basic understanding of meiosis but also for plant breeding applications.
> Thank you for your positive evaluation of the work.

There is only concern I have, if it comes to practical applications. Mutation of the well characterized CO suppressors RecQ4A or FancM results in somatic repair defects and hyperrecombination, which might cause unwanted secondary mutations. As far as I understand from the manuscript (I might be wrong) CTF18, SGO2, and SPF2 seem to have also important roles in somatic cells. I think the authors should elaborate on this more clearly in the discussion. The authors analyzed meiotic chromosome spreads to detect aberrations and genetic instability. It would be interesting to do the same with mitotic spreads, at least for the factors that are not meiosis-specific.

> This is an important point. We would like to mention that the suggested usage in breeding of these mutations does not imply that the mutations will be present in the commercial varieties. Indeed, increasing crossover in pericentromeric regions would be useful to introgress traits from non-elite source (e.g. related wild-species) into elite varieties. This occurs in the early phase of a breeding scheme, and the mutation(s), after having allowed the desired recombination events, will be likely segregated out together with undesired genomic regions from the source, and thus not present in the final varieties and thus not in the fields. In this context, a reasonable penalty on genome stability associated with the mutation is not problematic for its usage in breeding. This has been confirmed in discussions between the senior author of this study and several plant breeders. We added these considerations in the discussion of the revised manuscript.

Further, the Arabidopsis mutants *sgo2*, *spf2* and *ctf18* appear to be unaffected in growth and development (figure S2) and fertility (Figure 6). Admittedly, this does not exclude a possible penalty in crops under field or harsh conditions, but again plant breeding scheme would most likely not keep the mutation in the elite lines.
